# Annexin A1 exacerbates islet stellate cell activation by regulating triglyceride catabolism via the PPARα/ACOX1/CYP4a pathway

**DOI:** 10.1080/19382014.2026.2633793

**Published:** 2026-02-22

**Authors:** Qing Li, Yan Zhang, Tianpei Yu, Li Shi, Qinqin Qiu, Ben Wang, Yiquan Sang, Rui Li, Qian Lv, Jie Wang, Xuekui Liu, Houfa Geng, Peter M. Jones, Jun Liang, Wei Xu

**Affiliations:** a Department of Endocrinology, Affiliated Xuzhou Central Hospital of Southeast University, The Xuzhou Clinical College of Xuzhou Medical University, Xuzhou Clinical School of Nanjing Medical University, Xuzhou Central Hospital, Xuzhou, People's Republic of China; b Department of Endocrinology, Luxian People's Hospital, Luzhou, People's Republic of China; c Diabetes & Obesity, School of Cardiovascular and Metabolic Medicine & Sciences, King's College London, London, UK

**Keywords:** Islet stellate cells, lipid metabolism, ANXA1, PPARα, Islet fibrosis

## Abstract

**Background:**

Activation of islet stellate cells (ISCs) contributes to islet fibrosis and diabetes progression through excessive extracellular matrix secretion and lipid loss. Annexin A1 (ANXA1) has been reported to modulate lipid metabolism in other tissues, but its role in ISCs remains unclear.

**Methods:**

ISCs were isolated from 9-and 28-week-old db/m and db/db mice. Lipid content analysis, qRT‒PCR, and Western blotting were used to assess lipid metabolism-related molecules. ANXA1 expression was analyzed by immunohistochemistry and Western blotting. Recombinant ANXA1 was co-cultured with db/db ISCs to evaluate lipid synthesis and lipolysis. The interaction between ANXA1 and peroxisome proliferator-activated receptor alpha (PPARα) was examined by immunoprecipitation.

**Results:**

Activation of ISCs markedly reduced intracellular triglycerides, with decreased Diacylglycerol Acyltransferase 1/2 (DGAT1/2) and increased adipose triglyceride lipase (ATGL) and hormone-sensitive triglyceride lipase (HSL) expression. ANXA1 was detected in islets, MIN6 cells, and their culture supernatants. Recombinant ANXA1 treatment lowered triglyceride levels and upregulated PPARα and its downstream genes, acyl-CoA oxidase 1 (ACOX1) and cytochrome P450 4 A (CYP4A); these effects were enhanced by a PPARα agonist but reversed by inhibition. Immunofluorescence and coimmunoprecipitation confirmed that PPARα acts as a key mediator of ANXA1-regulated triglyceride metabolism in ISCs.

**Conclusion:**

ANXA1 promotes ISCs activation by enhancing triglyceride catabolism through the PPARα signaling pathway, suggesting a novel therapeutic target for islet fibrosis.

## Graphical abstract

**Figure:**
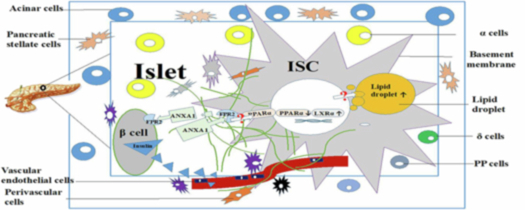

## Introduction

Islet fibrosis is a critical pathological feature of type 2 diabetes mellitus (T2DM) and directly contributes to *β*-cells dysfunction by disrupting the islet microenvironment.^1^ Following the onset of fibrosis, pancreatic islets are characterized by excessive deposition of extracellular matrix (ECM), destruction of normal islet architecture, a reduction in cellularity, and distortion of the microvascular network. These microcirculatory abnormalities impair islet blood supply and interfere with the normal secretion of islet endocrine hormones, thereby further exacerbating islet dysfunction.^2^^,^^3^ Therefore, a comprehensive understanding of the initiation, progression, and regulatory mechanisms of islet fibrosis has become a major focus of current research.

Our group first identified and defined islet stellate cells (ISCs). Similar to pancreatic stellate cells (PSCs), ISCs exist in either a quiescent or an activated state. Quiescent ISCs are characterized by abundant intracellular lipid droplets;^4^ however, upon exposure to external stimuli such as oxidative stress and inflammatory cytokines, they undergo activation, as evidenced by lipid loss, *α*-smooth muscle actin (*α*-SMA) expression, and enhanced migratory and proliferative capacities.^5^^,^^6^ Activated ISCs markedly increase ECM deposition within islets, thereby promoting the development of islet fibrosis.^7^ Accumulating evidence indicates that fatty acid metabolism plays a crucial role in ISCs activation.^8^ Previous studies on PSCs and hepatic stellate cells (HSCs) have demonstrated that reducing intracellular triglyceride accumulation promotes stellate cells activation and accelerates the progression of pancreatic and hepatic fibrosis.^9-12^ Inhibition of diacylglycerol acyltransferase 1 and 2 (DGAT1 and DGAT2), key enzymes involved in triglyceride synthesis, has been shown to enhance HSCs activation in mouse models of metabolic dysfunction-associated fatty liver disease.^13^ Conversely, deficiency of lipid-hydrolyzing enzymes such as adipose triglyceride lipase (ATGL) and hormone-sensitive lipase (HSL) leads to the accumulation of triglycerides and retinyl esters in HSCs,^14^ thereby attenuating their activation. However, studies focusing on the regulation of fatty acid metabolism in ISCs and its role in ISCs activation remain limited.

Annexin A1 (ANXA1) has been identified as an important endogenous anti-inflammatory protein that participates in various cellular processes, including inflammatory resolution, cell differentiation and proliferation, regulation of cell death signaling, and the clearance of apoptotic cells.^15^ Previous investigations of ANXA1 have primarily focused on inflammation, cancer, and cardiovascular diseases.^16^ Emerging evidence suggests that ANXA1 modulates lipid catabolism by regulating key lipolytic proteins, including ATGL, HSL, and galectin-12 (Gal-12).^17^ Moreover, ANXA1 has been shown to reduce lipid accumulation and delay the progression of hepatic fibrosis in non-alcoholic fatty liver disease.^18^ In ANXA1-deficient mice, the biosynthesis of unsaturated fatty acids, linoleic acid metabolism, and overall fatty acid biosynthesis are markedly suppressed.^19^

Peroxisome proliferator-activated receptor alpha (PPARα), a member of the nuclear hormone receptor superfamily, functions as a lipid sensor and plays a central role in regulating fatty acid transport, esterification, and oxidation.^20^^,^^21^ PPARα is a key regulator of fatty acid oxidation, and both global and liver-specific PPARα deficiency result in spontaneous hepatic steatosis and hyperlipidemia in mice fed a normal diet, highlighting its essential role in hepatic lipid metabolism.^22^ Chen et al. reported that targeted activation of PPARα promotes HSC activation, thereby contributing to liver fibrosis.^21^ Similarly, Dong et al. demonstrated that sirtuin 6 (SIRT6) enhances fatty acid oxidation by activating PPARα, reduces intracellular lipid accumulation in HSCs, and consequently promotes HSCs activation.^23^

Wu et al. further demonstrated that ANXA1 regulates mitochondrial fatty acid oxidation via the AMPK/PPARα/CPT1b signaling pathway, thereby alleviating lipid accumulation in renal tubular epithelial cells and improving diabetic nephropathy.^24^ However, whether ANXA1 regulates fatty acid metabolism and activation of ISCs through PPARα signaling remains unclear.

This study investigates the regulatory role of ANXA1 in lipid metabolism during the activation of ISCs and evaluates its potential as a therapeutic target for diabetes by providing new theoretical insights into its interaction with PPARα signaling pathways.

## Materials and methods

### Materials

#### Experimental animals

The animal experiments received approval from the Animal Ethics Committee of Xuzhou Medical University (Approval No.: SBE 2019740421; date: October 10, 2022), in accordance with the Guidance on the operation of the Animals (Scientific Procedures) Act 1986. Diabetic db/db mice (C57BL/KsJ-db/db background, 9 and 28 weeks old) and wild-type db/m control mice (C57BL/Ksj-db/m background) were purchased from the Nanjing University and housed under specific-pathogen-free (SPF) conditions at the Animal Experiment Center of Xuzhou Medical University.

### Methods

#### Islet stellate cell isolation

db/db mice (*n* = 10) and db/m mice (*n* = 10) were sacrificed through cervical dislocation, followed by cessation of circulation via the cutting of the femoral artery. The procedure for isolating islets was performed as previously described.^6^ Islets were isolated from db/m and db/db mice (9 weeks old, with 10 mice in each group, where blood glucose levels exceeded 13 mmol/l) by digesting the exocrine pancreas using type IV collagenase (1 mg/ml; Sigma, CA, USA) and subsequently purified on Histopaque 1077 (Sigma, CA, USA) density gradients. Purified islets were cultured in RPMI 1640 medium (Gibco, 6123120, USA) supplemented with 10% fetal bovine serum. After 1–2 d, star-shaped quiescent ISCs could be observed surrounding the islets. “Passage 0” is defined as 10 to 14 d after the islets were placed in culture, at which point the cultures were nearly confluent with stellate cells. Beginning at passage 0, cells were harvested using trypsin and subcultured (1:2) every 3–4 d. The cells were maintained in Dulbecco's modified Eagle's medium (DMEM)/F12 (1:1 v/v) (Sigma, CA, USA) containing 10% (v/v) fetal bovine serum (FBS) and were utilized from passage 3–8.

#### Cultivation and intervention of MIN6 cells

The MIN6 mouse pancreatic *β*-cells line used in this experiment was purchased from Suzhou Haixing Biological Co., Ltd. in China. Control medium: supplemented with 15% fetal bovine serum, 1% glutamine, and 1% penicillin/streptomycin. Experimental group: DMEM/F12 + 10% fetal bovine serum + 1% glutamine + 1% penicillin/streptomycin + high glucose (25  mmol/L). Specific culture method: After thawing, the cells were placed in a 37 °C, 5% CO₂ incubator. Passaging was performed when cells confluence reached approximately 80%, as observed under a microscope.

#### Oil Red O staining

ISCs were cultured in condition medium or in a medium supplemented with rhANXA1 (100 ng/ml) for 72 h unless otherwise specified. The samples were fixed in 4% PFA in PBS for 20 min at room temperature. Oil red O (Nanjing Punuoen Technology, PDL001, China) staining was performed by incubating PFA-fixed material for 10 min a saturated solution of Oil red O in isopropanol, followed by washing with distilled water. Images were taken using a phase contrast microscopy (Zeiss).^5^

#### Cholesterol and triglyceride assay for ISCs

The tissue lipid content assay kit (Beijing Pulilai, E1013, E1015) was used to measure the intracellular cholesterol and triglyceride levels. ISCs pellets were collected, and 0.1 ml of lysis buffer was added for every 1 × 10⁶ cells. After mixing and incubating at room temperature for 10 min, the supernatant was transferred to 1.5 mL centrifuge tubes, heated to 70 °C for 10 min, and then centrifuged at 2000 rpm for 5 min. The clear upper layer was used for enzymatic assays. Each well received a 10 µl sample and was incubated at 37 °C for 15 min. OD values were measured using a microplate reader after the blank was zeroed.

#### Immunohistochemistry

Immunohistochemistry was performed as previously described.^25^ The pancreas from 9- and 28-week-old mice (*n* = 3) was perfused and fixed in 4% paraformaldehyde in 0.1 M PBS at 4 °C for 24 h, embedded in paraffin, and sectioned to a thickness of 4 µm. The paraffin sections were blocked with 5% BSA for 30 min, incubated overnight at 4 °C with rabbit anti-mouse ANXA1 antibody (1:200, Abcam, Cambridge, UK), washed, and then incubated at room temperature with HRP-conjugated anti-rabbit IgG for 30 min. The slides were developed using DAB, counterstained with hematoxylin, and examined with an Olympus BX40 microscope. For immunofluorescence, cells were washed with TBS, fixed with 4% neutral formaldehyde at 4 °C for 15 min, and stained with DAPI. Sections were processed with dewaxing, hydration, antigen retrieval, and staining. Fluorescence images were acquired using a fluorescence microscope.

#### Quantitative real-time PCR (qRT–PCR)

ISCs were seeded in Nunclon™ 35 mm Petri dishes and cultured in condition medium, with rhANXA1 (100 ng/ml) or with FRP2 shRNAs for 48 h unless otherwise specified. qRT‒PCR was conducted using the SYBR® Green real-time PCR kit (TaKaRa BIO, Otsu, Japan) on the ABI StepOnePlus Real-Time PCR system (Applied Biosystems, Foster City, CA, USA).^6^ All quantifications were performed using U6 as the internal standard. The PCR primer sequences can be found in Supplementary Document 2 (S2).

#### Western blotting analysis

MIN6 cells were cultured in condition medium or in medium supplemented with D-glucose (25 mmol/l) for 48 h, unless otherwise stated. ISCs were seeded into Nunclon™ 35 mm Petri dishes and cultured in condition medium or in the presence of rhANXA1 (Proteintech, 21990- 1AP, 100 ng/mL) for 72 h, or with rhANXA1 plus PPARα agonist (GW9578, 5 nM, 30 h, MCE, USA), or rhANXA1 plus PPARα antagonist (GW6471, 25 µM, 30 h, MCE, CA, USA), or with FPR2 shRNAs for 48 h, unless otherwise stated.

Western blotting was performed using the following primary antibodies: ANXA1, PPARα, DGAT1, DGAT2, ATGL, HSL, CPT1b, ACOX1, CYP4A1, mouse IgG, and rabbit IgG. Annexin A1 Polyclonal antibody (1:5000, 21990-1-AP, Proteintech); PPARα Monoclonal antibody (1:2000, 66826-1-Ig, Proteintech); DGAT1 Rabbit mAb (1:500, A23077); DGAT2 Polyclonal antibody (1:500, 17100-1-AP, Abclonal); ATGL Polyclonal antibody (1:500, 55190-1-AP, Proteintech); HSL Polyclonal antibody (1:4000, 17333-1-AP, Proteintech); CPT1B-specific Polyclonal antibody (1:4000, 22170-1-AP, Proteintech); ACOX1 Polyclonal antibody (1:4000, 10957-1-AP, Proteintech); CYP4A1 Polyclonal antibody (1:200, 18054-1-AP, Proteintech); mouse IgG (A7028, Beyotime Biotech Inc.); and rabbit IgG (A7016, Beyotime Biotech Inc.). Blot/gel image data are provided in Supporting Information 1 (S1).

#### shRNA preparation and targeting gene knockdowns

Specific shRNAs and control shRNA were designed and synthesized by HANBIO (Shanghai, China). A BLAST search was conducted using the National Center for Biotechnology Information (NCBI) database to ensure that the shRNA constructs targeted only mouse FPR2/ALX. To knock down mouse FPR2/ALX in ISCs, FPR2/ALX shRNA (1 × 10^7^TU/ml) (RiboBio Co., Ltd.) was administered to the experimental group, while the corresponding negative control shRNA was transfected into the control group.

#### Immunoprecipitation (IP)

According to the operating instructions, coimmunoprecipitation assays were performed in ISCs using the Pierce Classic Magnetic IP/Co-IP Kit (ThermoFisher Scientific, USA). In brief, cold IP lysis was employed to lyse the cells. Five hundred micrograms of protein were obtained following the protein extraction procedure and mixed with 500 µL of IP lysate. A total of 5 µg of ANXA1 or PPARα antibody were utilized to incubate the protein, forming an antigen-antibody complex. Protein A/G magnetic beads and the antigen- antibody complexes were combined and incubated at room temperature for 1 h. After thorough washing, the antigen-antibody complexes were eluted using an elution buffer. Finally, the products after elution were subjected to western blotting analysis to detect PPARα or ANXA1 protein.

#### Statistical analysis

Data were analyzed using R software (version 4.2.1). Independent samples t tests or ANOVA were performed. Repeated-measures ANOVA with Bonferroni post hoc tests examined measurements across different time points. A *P*-value < 0.05 was regarded as statistically significant.

## Results

### Differences in lipid metabolism between db/m ISCs and db/db ISCs

**Figure 1.**
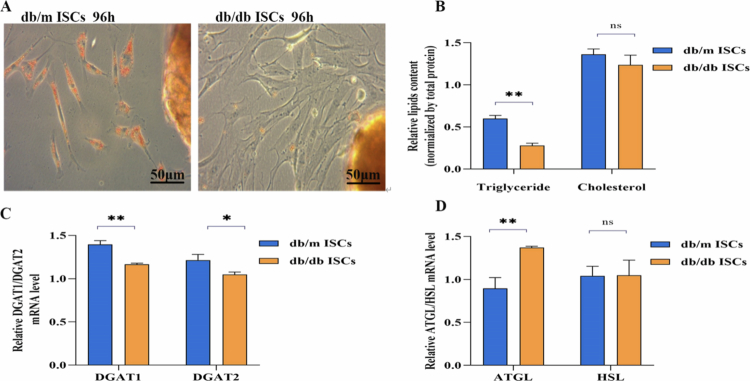
Differences in lipid metabolism between db/m ISCs and db/db ISCs. A: Oil red “O” staining of lipid droplets in cytoplasm from ISCs shows faster activation and loss of lipid droplets in diabetic ISCs. Scale bar = 50 μm. B: The tissue lipid content assay indicated that the triglyceride content decresead in db/db ISCs; there was no difference in cholesterol levels between the two groups of cells. Data are expressed as mean ± SE (*n* = 3), **P < 0.01 vs db/m ISCs group; The *P* values were calculated using two-way ANOVA with the Bonferroni post hoc test. All analyzes were conducted in triplicate. C: qRT‒PCR analyzes of ISCs were conducted to measure DGAT1 and DGAT2 mRNA levels. qRT‒PCR showed that compared with db/m ISCs, the activities of lipase DGAT1 and DGAT2 in db/db ISCs decreased. Data are presented as means ± SE (*n* = 3); *P < 0.05, **P < 0.01 vs db/m ISCs group; the *P* values were calculated using two-way ANOVA with the Bonferroni post hoc test. All qRT‒PCR analyzes were performed three times. D: qRT‒PCR analyzes of ISCs were conducted to measure ATGL and HSL mRNA levels. qRT‒PCR showed that compared with db/m ISCs, the activities of lipase DGAT1 and DGAT2 in db/db ISCs increased. Data are presented as means ± SE (*n* = 3); *P < 0.05, **P < 0.01 vs db/m ISCs group; The *P* values were calculated using two-way ANOVA with the Bonferroni post hoc test. All qRT‒PCR analyzes were performed three times.

Oil red staining revealed that the red lipids in db/db ISCs disappeared significantly more quickly than those in db/m ISCs (Figure 1A). The lipid content assay showed that the intracellular triglyceride content in db/db ISCs was significantly lower than in db/m ISCs, while there was no significant difference in cholesterol content between the two groups (Figure 1B). qRT–PCR results indicated that, compared with db/m ISCs, the activities of lipases DGAT1 and DGAT2 decreased in db/db ISCs (Figure 1C), whereas the activities of lipases ATGL and HSL increased (Figure 1D). In summary, the notable changes in triglycerides may play a crucial role in activating ISCs. Future studies will focus on triglyceride metabolism in ISCs.

### ANXA1 protein and its receptor FPR2 expression in islets, MIN6 and ISCs

**Figure 2.**
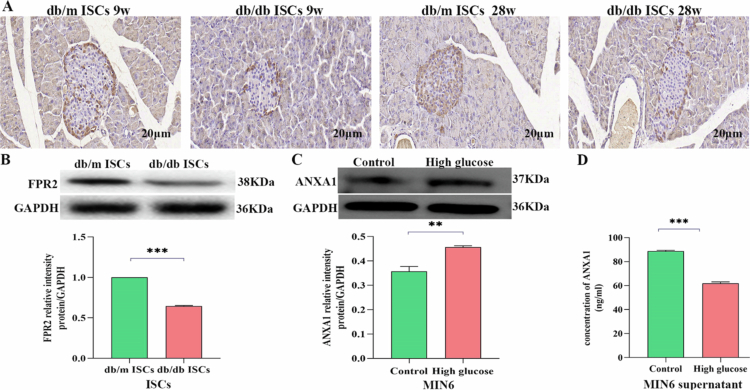
ANXA1 protein and its receptor FPR2 expression in islets, MIN6 cells and ISCs. A: Wax-embedded sections of db/m and db/db mouse pancreases, aged 9 and 28 weeks, demonstrating the expression of ANXA1 via immunohistochemistry. Scale bar = 20 μm. All analyzes were performed three times. B: Western blotting of ISCs using FPR2 antibody. Data are expressed as mean ± SE (*n* = 3); *P < 0.05, ***P < 0.001 vs db/m ISCs group. The *P* values were calculated using two-way ANOVA with the Bonferroni post hoc test. All analyzes were conducted in triplicate. C: Western blotting of MIN6 cells using ANXA1antibody. Data are expressed as mean ± SE (*n* = 3); *P < 0.05, **P < 0.01 vs Con group. The *P* values were calculated using two-way ANOVA with the Bonferroni post hoc test. All analyzes were conducted in triplicate. D: Western blotting of the culture supernatant of MIN6 cells using ANXA1antibody. Data are expressed as mean ± SE (*n* = 3); *P < 0.05, ***P < 0.001 vs Con group. The *P* values were calculated using two-way ANOVA with the Bonferroni post hoc test. All analyzes were conducted in triplicate.

Figure 2A shows that ANXA1 protein is expressed in the pancreatic tissues of both db/m and db/db mice. Compared with db/m mice, the ANXA1 protein in the pancreatic tissues of db/db mice is significantly reduced, and it decreases further with age. Figure 2B shows that the pancreatic stellate cells of both db/m and db/db mice express FPR2 receptors, and the protein expression level of FPR2 in db/db ISCs is significantly lower than in db/m ISCs. To evaluate the expression of ANXA1 protein in MIN6 cells and their culture supernatant, we used Western blotting for verification. As shown in Figure 2C, compared with the control group, the level of ANXA1 protein in MIN6 cells increased under high glucose conditions (25 mmol/L) (*P* < 0.01). Figure 2D shows that the expression of ANXA1 protein in the culture supernatant of MIN6 cells was significantly lower than that in the control group (*P* < 0.001). The data above indicate that ANXA1 protein is expressed in the pancreas, MIN6 cells, and their culture supernatant, which may influence the biological functions of adjacent ISCs within the pancreatic islet microenvironment.

### shRNA (FPR2) transfection and characterization

**Figure 3.**
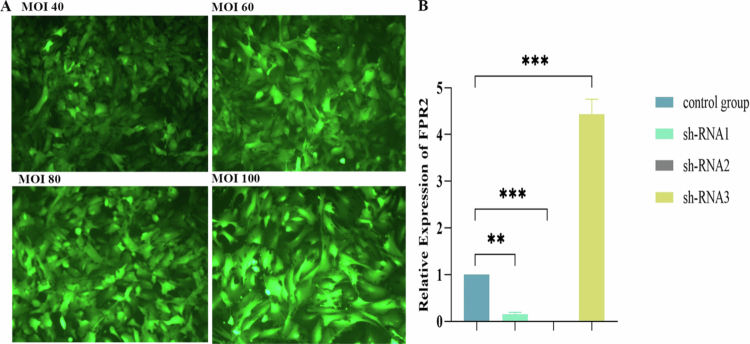
Identification of sh-RNA transfection effect. A: Fluorescence effect of sh-RNA lentiviral transfection, MOI value = viral titer (TU/mL) × viral volume (mL)/number of cells; B: qRT‒PCR analyzes of db/db ISCs were conducted to measure FPR2 mRNA levels. qRT‒PCR showed that compared with sh-RNA1 and sh-RNA3, the activities of FPR2 in sh-RNA2 group decreased significantly. Data are presented as mean ± SE (*n* = 3); *P < 0.05, ***P < 0.001 vs sh-RNA1 group; The *P* values were calculated using two-way ANOVA with the Bonferroni post hoc test. All qRT‒PCR analyzes were performed three times.

As shown in Figure 3A, the transfection efficiency of ISCs from db/db diabetic mice was greater than 90% after 48 h of transfection using lentivirus. As shown in Figure 3B, FPR2 receptor expression after lentiviral transfection was observed by qRT‒PCR experiments, and shRNA2 was selected for subsequent experiments.

### Effect of ANXA1 on triglycerides and fatty acid synthase, and fatty acid catabolism enzymes

**Figure 4.**
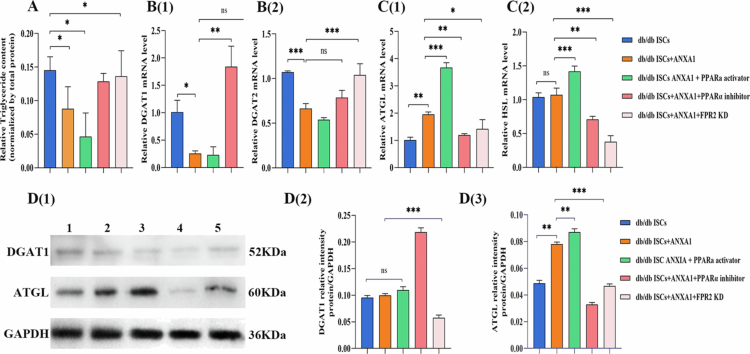
Effect of ANXA1 on triglycerides and fatty acid synthase, and fatty acid catabolism enzymes. A: Differences in triglyceride content among the groups; the results showed that after the addition of exogenous ANXA1 protein (100 ng/mL), the intracellular triglyceride in the db/db ISCs was significantly reduced. Data are presented as means ± SE (*n* = 3); *P < 0.05, **P < 0.01 vs db/db ISCs group; The *P* values were calculated using two-way ANOVA with the Bonferroni post hoc test. B: qRT‒PCR analyzes of ISCs were conducted to measure DGAT1 and DGAT2 mRNA levels in each group after addition of exogenous ANXA1 protein, PPARα receptor agonist, PPARα receptor inhibitor, and lentiviral knockdown of FPR2 receptor; Data are presented as mean ± SE (*n* = 3); *P < 0.05, **P < 0.01, ***P < 0.001 vs db/db ISCs group; The *P* values were calculated using two-way ANOVA with the Bonferroni post hoc test. All qRT‒PCR analyzes were performed three times. C: qRT‒PCR analyzes of ISCs were conducted to measure ATGL and HSL mRNA levels in each group after addition of exogenous ANXA1 protein, PPARα receptor agonist, PPARα receptor inhibitor, and lentiviral knockdown of FPR2 receptor; data are presented as mean ± SE (*n* = 3); *P < 0.05, **P < 0.01, ***P < 0.001 vs db/db ISCs group; The *P* values were calculated using two-way ANOVA with the Bonferroni post hoc test. All qRT‒PCR analyzes were performed three times. D: Quantitative analysis of the results of Western blotting experiments. PPARα activator: PPARα receptor agonist; PPARα inhibitor: PPARα receptor inhibitor; FPR2 KD: knockdown of FPR2 receptor; data are presented as the mean ± SE (*n* = 3); *P < 0.05, **P < 0.01, ***P < 0.001 vs the db/db ISCs group; The *P* values were calculated using two-way ANOVA with the Bonferroni post hoc test. All qRT‒PCR analyzes were performed three times.

To evaluate the impact of ANXA1 protein on triglyceride metabolism, we assessed the expression levels of intracellular triglyceride storage, fatty acid synthase, and fatty acid degradation-related genes and proteins via qRT-PCR and Western blotting. Results showed that exogenous ANXA1 protein (100 ng/mL) significantly reduced intracellular triglycerides in db/db mouse adipose stem cells (Figure 4A). Transcriptional levels of fatty acid synthases DGAT1 and DGAT2 decreased (Figure 4B), while lipase ATGL transcription increased (Figure 4C). Addition of the PPARα agonist (GW9578, 5 nM) further reduced DGAT2 transcription (Figure 4B) and significantly increased ATGL and HSL transcription (Figure 4C); addition of the PPARα antagonist (GW6471, 25 μM) decreased ATGL and HSL transcription (Figure 4B and C). The Western blotting results showed that, following the addition of exogenous ANXA1 protein, the expression of the fat synthesis-related enzyme DGAT1 decreased, while the expression of the lipolytic enzyme ATGL increased. db/db ISCs DGAT1 expression was further decreased, and ATGL expression was further increased by the addition of PPARα agonist compared with the db/db ISCs + ANXA1 protein group; after the addition of PPARα inhibitor, the expression of db/db ISCs DGAT1 increased, and ATGL expression further decreased (Figure 4D).

### Effects and mechanism of ANXA1 on triglyceride metabolism in db/db ISCs

**Figure 5.**
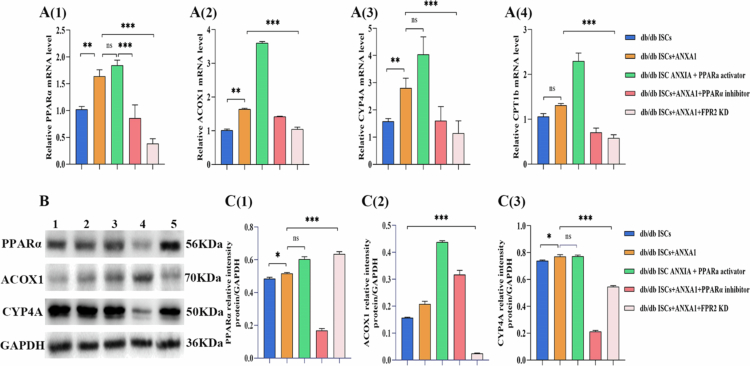
The effect of ANXA1 on triglyceride metabolism and its underlying mechanism. A: qRT‒PCR analyzes of ISCs were conducted to measure PPARα and its downstream genes ACOX1, CPT1b, and CYP4a mRNA levels in each group. *P < 0.05, **P < 0.01, ***P < 0.001 vs db/db ISCs group; The *P* values were calculated using two-way ANOVA with the Bonferroni post hoc test. All qRT‒PCR analyzes were performed three times. B: Western blotting of PPAR *α* and its downstream genes ACOX1, CPT1b, and CYP4a; C: Quantitative protein analysis of PPAR *α* and its downstream genes ACOX1, CPT1b, and CYP4a; 1: db/db ISCs; 2: db/db ISCs + ANXA1; 3: db/db ISCs + ANXA1 + PPARα activator; 4: db/db ISCs + ANXA1 + PPARα inhibitor; 5: db/db ISCs + ANXA1 + FPR2 KD. *P < 0.05, **P < 0.01, ***P < 0.001. The *P* values were calculated using two-way ANOVA with the Bonferroni post hoc test. All qRT‒PCR analyzes were performed three times.

Intracellular triglycerides, ACOX1, CPT1b, and CYP4A genes downstream of PPARα were measured after the addition of exogenous ANXA1 protein. As shown in Figure 5, the expression of PPARα and its downstream genes ACOX1, CPT1b, and CYP4A genes was increased after the addition of exogenous ANXA1 protein; the results again suggested that the ANXA1 protein promotes triglyceride catabolism in db/db ISCs may act through the PPARα signaling pathway. Further validation of the involvement of PPARα and downstream genes in db/db ISCs triglyceride metabolism was performed with PPARα agonists and PPARα inhibitors, and the results are shown in Figure 5B. Compared with the db/db ISCs + ANXA1 protein group, addition of PPARα agonists resulted in a reduction of db/db ISCs triglycerides and an increase in the expression of downstream genes ACOX1, CYP4A, and CPT1b. After adding PPARα inhibitor, db/db ISCs triglycerides increased, and downstream genes ACOX1, CYP4A, and CPT1b expression decreased, which above reconfirmed that ANXA1 protein can promote db/db ISCs triglyceride catabolism by activating PPARα downstream genes ACOX1, CPT1b, and CYP4A. The western blotting results showed that the expression of PPARα and its downstream ACOX1, CYP4A was increased by the addition of ANXA1 protein compared with the db/db ISCs group. Compared with the db/db ISCs + ANXA1 protein group, downstream ACOX1 and CYP4A expression was increased by the addition of PPARα agonist, and downstream ACOX1 and CYP4A expression was decreased by the addition of PPARα inhibitor in db/db ISCs (Figure 5C), which was in agreement with the qRT‒PCR results. To find out whether ANXA1 protein acts through its receptor FPR2, the FPR2 receptor on the surface of db/db ISCs was silenced by lentiviral transfection, and it was found that, compared with the group of db/db ISCs + ANXA1 protein, the promotional effect of ANXA1 protein on the expression of PPARα gene disappeared when the FPR2 receptor was knocked down. qRT–PCR suggested that ANXA1 protein exerts effects on ISCs triglyceride metabolic pathway through its receptor FPR2 (Figure 5B). Western blotting results showed that compared with the group with db/db ISCs + ANXA1 proteins, after knocking out the FPR2 receptor, the expression level of PPARα proteins increased (Figure 5C).

### Relationship between ANXA1 protein and PPARα

**Figure 6.**
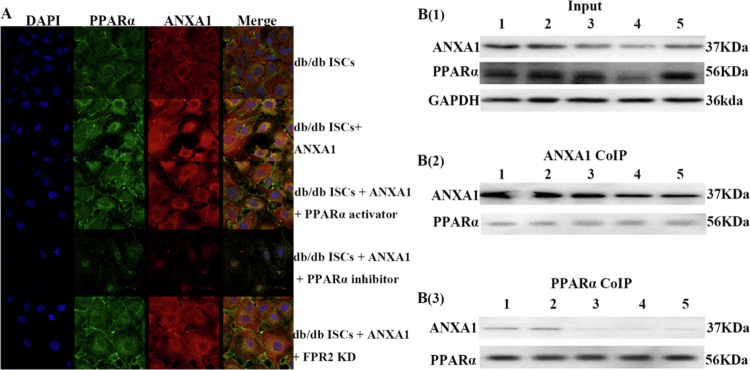
Relationship between ANXA1 protein and PPARα. A: Immunofluorescence analysis showed that the expression of PPARα was increased by adding exogenous ANXA1 protein. Silencing of FPR2 further increased PPARα expression, scale bar = 20 μm. B: Coimmunoprecipitation (coIP) analysis showed the expression of PPARα in each experimental group and confirmed the binding between ANXA1 and PPARα. PPARα activator: PPARα receptor agonist; PPARα inhibitor: PPARα receptor inhibitor; FPR2 KD: Knockdown of FPR2 receptor; 1: db/db ISCs; 2: db/db ISCs + ANXA1; 3: db/db ISCs + ANXA1 + PPARα. + ANXA1 + PPARα activator; 4: db/db ISCs + ANXA1 + PPARα inhibitor; 5: db/db ISCs + ANXA1 + FPR2 KD.

The co-IP assays revealed a possible protein–protein interaction between ANXA1 and PPARα (Figure 6). Immunofluorescence analysis showed an increase in PPARα expression in response to exogenous ANXA1 protein, along with further increases in PPARα expression following FPR2 silencing (Figure 6A). These findings suggest that ANXA1 affects its actions through interactions with PPARα, potentially mediated by FPR2 (Figure 6B). *P < 0.05, **P < 0.01, ***P < 0.001.

## Discussion

Our study demonstrates that ANXA1 activates islet stellate cells by modulating triglyceride metabolism, mainly through activating PPARα and its downstream genes. When exogenous ANXA1 protein is added, intracellular triglyceride levels in ISCs decrease rapidly, while the expression of PPARα and its downstream genes ACOX1, CPT1b, and CYP4A increases. Moreover, the expression of lipid synthesis enzymes DGAT1 and DGAT2 declines, whereas lipid lipase enzymes ATGL and HSL increase. This results in lower intracellular triglycerides and facilitates the transition of ISCs from a resting to an active state. This is the first study to investigate the link between ANXA1 and lipid metabolism in ISCs.

In this study, we compared the lipid content in ISCs under two different states using Oil Red O staining. The results showed that the disappearance rate of red lipid droplets in db/db ISCs was significantly faster than in db/m ISCs, consistent with previous studies, further confirming the link between lipid metabolism and ISC activation. Additionally, compared to db/m ISCs, triglyceride and cholesterol levels were lower in db/db ISCs, with significant statistical differences observed in triglyceride changes. The expression of lipid synthesis-related enzymes DGAT1 and DGAT2 was decreased, while the expression of lipid breakdown-related enzymes ATGL and HSL was increased. These findings suggest that alterations in triglyceride content play a crucial role in the activation of ISCs, which is consistent with previous studies on lipid metabolism in other stellate cells,^26^ confirming that a reduction in intracellular triglyceride levels is associated with ISCs activation.

Interestingly, we observed a decrease in ANXA1 protein levels in the supernatant of pancreatic islets from 28-week-old diabetic mice, while ANXA1 protein levels increased in MIN6 cells cultured under high-glucose conditions. We hypothesize that the difference in ANXA1 protein levels in MIN6 cells from diabetic mice and those cultured under high-glucose conditions may reflect a protective mechanism in *β*-cells against short-term high-glucose exposure. During short-term high glucose exposure, *β-*cells produce more ANXA1 to maintain insulin secretion. However, over time, the sustained high secretion gradually diminishes, leading to a decrease in ANXA1 levels. The reduction of ANXA1 in the supernatant of MIN6 cells under high-glucose conditions might also suggest an autoprotective mechanism. We propose that, in the diabetic islet microenvironment, *β-*cells may reduce paracrine ANXA1 protein to attenuate the stimulatory effect on nearby ISCs, serving as a protective response to delay the progression of islet fibrosis.

Studies have confirmed an association between ANXA1 and PPARα,^27^ which may be a key factor in regulating ANXA1-mediated lipid metabolism. Building on this, the present study further explored the interaction between ANXA1 and PPARα in ISCs. Immunofluorescence co-localization and co-immunoprecipitation experiments validated the binding of ANXA1 to PPARα in ISCs. Additionally, q-RTPCR, Western blotting, and immunofluorescence results indicated that following the exogenous addition of ANXA1 protein, the expression of PPARα was significantly upregulated, suggesting that PPARα is a crucial factor for ANXA1's regulatory effects. Therefore, we hypothesize that ANXA1 may regulate triglyceride metabolism in ISCs via PPARα, thereby influencing islet fibrosis and islet function.

To further investigate the specific molecular mechanisms by which ANXA1 influences triglyceride metabolism in ISCs, we focused on the classic lipid metabolism pathway PPARα/CPT1b/ACOX1/CYP4A.^28^ Following the addition of exogenous ANXA1 protein, the expression of PPARα and its downstream genes and proteins (CPT1b, ACOX1, CYP4A) increased. Intervention with the PPARα agonist GW6471 further elevated the expression of these genes and proteins. Conversely, after intervention with a PPARα inhibitor, the expression of CPT1b, ACOX1, and CYP4A genes and proteins decreased, reversing the effects of ANXA1 protein on ISCs. These results confirm that ANXA1 may promote the breakdown of intracellular triglycerides in ISCs and accelerate their activation, leading to islet dysfunction by activating PPARα and its downstream genes.

In studies on alcoholic fatty liver, Schulze et al. demonstrated that downregulation of PPARα and its downstream genes ACOX1 and CPT1 reduces the rate of fatty acid *β*-oxidation, leading to hepatic lipid accumulation and liver dysfunction. However, the addition of a PPARα agonist can restore PPARα/RXRα DNA binding activity, induce mRNA expression of PPARα and its downstream genes ACOX and CPT1, stimulate fatty acid *β*-oxidation, and inhibit the development of alcoholic fatty liver.^29^ This finding is consistent with Wu Li et al.'s research and the outcomes of this study. All of these studies suggest that fatty acid metabolism can be regulated via the PPARα/ACOX/CPT1 pathway, but the final effects differ. Schulze RJ and Wu Li's studies have confirmed that activation of the PPARα/ACOX/CPT1 pathway enhances fatty acid metabolism, reducing lipid deposition in the liver and kidneys, and thereby improving liver and kidney function. In contrast, this study demonstrates that activation of the PPARα/ACOX/CPT1 pathway accelerates the rate of triglyceride disappearance in ISCs, leading to their activation and ultimately resulting in islet fibrosis. We speculate that this effect may be related to the different biological responses triggered by variations in gene expression across different target organs and cells. Moreover, Schulze RJ and Wu Li's studies focus on changes in the PPARα/ACOX/CPT1 signaling pathway after lipid deposition occurs in organs such as the liver and kidneys, whereas this study focuses on lipid metabolism within ISCs. A certain amount of triglycerides within the cell may be a key factor in maintaining the quiescent state of ISCs. Under the influence of specific regulatory factors, such as ANXA1 protein or others, the reduction of physiological levels of triglycerides within the cell can accelerate their activation.

In this study, q‒PCR and Western blotting revealed inconsistencies regarding whether the ANXA1 protein exerts its biological effects through its receptor, FPR2. In the qRT–PCR experimental results, compared with the group that only added exogenous ANXA1 protein, the knockout of the FPR2 receptor resulted in a decrease in the expression of PPARα and its downstream genes, ACOX1 and CPT1b, suggesting that ANXA1 protein can activate PPARα and its downstream genes through the FPR2 receptor. This result is inconsistent with the Western blotting experimental results. After repeated verification, we considered that the possible reason is that gene expression is divided into transcription and translation levels, with a temporal and spatial interval between the occurrence of transcription and translation in eukaryotic gene expression. For example, in the delayed effect, protein synthesis requires a certain amount of time, while mRNA expression can respond to stimuli more quickly, resulting in a time difference in changes and causing inconsistencies in expression at the mRNA and protein levels.^30^ Secondly, post-transcriptional processing is also divided into several levels, including the degradation of transcription products, translation, post-translational processing, and modification, which can also lead to inconsistencies between transcription and translation levels. For example, issues such as the stability of proteins after translation and modifications such as increased ubiquitination after protein translation, which can lead to protein degradation through the proteasome pathway, may all result in a decrease in protein levels.^31^ Both Western blotting and immunofluorescence results showed that after knocking out the FPR2 receptor, the expression of PPARα increased, indicating that the ANXA1 protein may regulate triglyceride metabolism through other receptor-independent pathways. The specific mechanism still needs to be further explored by us. Interestingly, we found that “low ANXA1-high TG“ results appear in clinical studies (data not published). The decrease of ANXA1 protein in the circulation of diabetic patients may be involved in the occurrence of hypertriglyceridaemia, while the decline of ANXA1 protein levels in the local islet high-glucose environment may reduce the triglyceride breakdown rate of ISCs, inhibit their activation, and thereby delay the progression of diabetic islet fibrosis. Therefore, both clinical and basic research indicate the dual role of ANXA1 protein in “systemic lipid metabolism“ and “local ISCs lipid homeostasis“. This study also has certain limitations. Our study lacks in vivo experimental data from ANXA1 gene knockout mouse models and therefore cannot confirm the effects and regulatory role of ANXA1 protein on ISCs activation at the in vivo level.

In conclusion, our data provide evidence that ANXA1 promotes the activation of islet stellate cells by regulating triglyceride catabolism through PPARα, offering a potential therapeutic target for diabetes. Future studies will further elucidate the molecular pathways influenced by ANXA1 in pancreatic islet fibrosis.

## Supplementary Material

supplementary materials Table 3 Instruments.docx supplementary materials Table 3: Instruments.docx

supplementary materials 4 Fig 6 quantitative analysis.tif supplementary materials 4: Fig 6 quantitative analysis.tif

supplementary materials_raw_images updated.pdf supplementary materials_raw_images updated.pdf

## Data Availability

Data will be made available on request.

## References

[1] Hu F, Qiu X, Bu S. Pancreatic islet dysfunction in type 2 diabetes mellitus. Arch Physiol Biochem. 2020;126(3):235–241. doi: 10.1080/13813455.2018.1510967. 30293453

[2] Haber PS, Keogh GW, Apte MV, Moran CS, Stewart NL, Crawford DH, Pirola RC, McCaughan GW, Ramm GA, Wilson JS. Activation of pancreatic stellate cells in human and experimental pancreatic fibrosis. Am J Pathol. 1999;155(4):1087–1095. doi: 10.1016/S0002-9440(10)65211-X. 10514391 PMC1867025

[3] Lee E, Ryu GR, Ko SH, Ahn YB, Yoon KH, Ha H, Song K. Antioxidant treatment May protect pancreatic beta cells through the attenuation of islet fibrosis in an animal model of type 2 diabetes. Biochem Biophys Res Commun. 2011;414(2):397–402. doi: 10.1016/j.bbrc.2011.09.087. 21971557

[4] Yang X, Chen J, Wang J, Ma S, Feng W, Wu Z, Guo Y, Zhou H, Mi W, Yin B, et al. Very-low-density lipoprotein receptor-enhanced lipid metabolism in pancreatic stellate cells promotes pancreatic fibrosis. Immunity. 2022;55(7):1185–1199. doi: 10.1016/j.immuni.2022.06.001. 35738281

[5] Zha M, Li F, Xu W, Chen B, Sun Z. Isolation and characterization of islet stellate cells in rat. Islets. 2014;6(2):e28701. doi: 10.4161/isl.28701. 25483957 PMC4594200

[6] Li FF, Chen BJ, Li W, Li L, Zha M, Zhou S, Bachem MG, Sun Z. Islet stellate cells isolated from fibrotic islet of goto-kakizaki rats affect biological behavior of beta-cell. J Diabetes Res. 2016;2016:6924593. doi: 10.1155/2016/6924593. 26697502 PMC4678093

[7] Wang X, Li W, Chen J, Zhao S, Qiu S, Yin H, Carvalho V, Zhou Y, Shi R, Hu J, et al. A transcriptional sequencing analysis of islet stellate cell and pancreatic stellate cell. J Diabetes Res. 2018;2018:7361684. doi: 10.1155/2018/7361684. 29619382 PMC5830286

[8] LI W, Zhou Y, WANG X, Cai M, Gao F, Carlsson P, Sun Z. Amodified in vitro tool for isolation and characterization of rat quiescent islet stellate cells. Exp Cell Res. 2019;384(1):111617. doi: 10.1016/j.yexcr.2019.111617. 31505166

[9] Sunami Y, Rebelo A, Kleeff J. Lipid metabolism, and lipid droplets in pancreatic cancer and stellate cells. Cancers (Basel). 2017;10(1):3. doi: 10.3390/cancers10010003. 29295482 PMC5789353

[10] Bansal SK, Bansal MB. Pathogenesis of MASLD and MASH - the role of insulinresistance and lipotoxicity. Aliment Pharmacol Ther. 2024;59(Suppl 1):S10–S22. doi: 10.1111/apt.17930. 38451123

[11] Jing XY, Yang XF, Qing K, Ou-Yang Y. Roles of the lipid metabolism in hepatic stellate cells activation △. Chin Med Sci J. 2013;28(4):233–236. doi: 10.1016/S1001-9294(14)60008-0. 24382226

[12] Vicente CP, Guaragna RM, Borojevic R. Lipid metabolism during in vitro induction of the lipocyte phenotype in hepatic stellate cells. Mol Cell Biochem. 1997;168(1-2):31–39. doi: 10.1023/A:1006845808305. 9062891

[13] Longo M, Paolini E, Di Benedetto P, Tomassini E, Meroni M, Dongiovanni P. DGAT1 and DGAT2 inhibitors for metabolic dysfunction-associated steatotic liver disease (MASLD) management: benefits for their single or combined application. Int J Mol Sci. 2024;25(16):9074. doi: 10.3390/ijms25169074. 39201759 PMC11354429

[14] Elinav E, Ali M, Bruck R, Brazowski E, Phillips A, Shapira Y, Katz M, Solomon G, Halpern Z, Gertler A. Competitive inhibition of leptin signaling results in amelioration of liver fibrosis through modulation of stellate cell function. Hepatology. 2009;49(1):278–286. doi: 10.1002/hep.22584. 19065677

[15] John CD, Christian HC, Morris JF, Flower RJ, Solito E, Buckingham JC. Annexin 1 and the regulation of endocrine function. Trends Endocrinol Metab. 2004;15(3):103–109. doi: 10.1016/j.tem.2004.02.001. 15046738

[16] Purvis GSD, Collino M, Loiola RA, Baragetti A, Chiazza F, Brovelli M, Sheikh MH, Collotta D, Cento A, Mastrocola R, et al. Identification of AnnexinA1 as an endogenous regulator of RhoA, and its role in the pathophysiology and experimental therapy of Type-2 diabetes. Front Immunol. 2019;10:571. doi: 10.3389/fimmu.2019.00571. 30972066 PMC6446914

[17] Dewhurst-Trigg R, Hopkinson J, Richardson S, Jones P, Rackham C. Mesenchymal stromal cells and their secretory products reduce the inflammatory crosstalk between islets and endothelial cells. Endocrine. 2025;87(1):94–105. doi: 10.1007/s12020-024-03975-1. 39085567 PMC11739262

[18] Rackham CL, Vargas AE, Hawkes RG, Amisten S, Persaud SJ, Austin AL, King AJ, Jones PM. Annexin A1 is a key modulator of mesenchymal stromal cell-mediated improvements in islet function. Diabetes. 2016;65(1):129–139. doi: 10.2337/db15-0990. 26470781

[19] Fan JH, Luo N, Liu GF, Xu XF, Li SQ, Lv XP. Mechanism of annexin A1/N-formylpeptide receptor regulation of macrophage function to inhibit hepatic stellate cell activation through Wnt/β-catenin pathway. World J Gastroenterol. 2023;29(22):3422–3439. doi: 10.3748/wjg.v29.i22.3422. 37389234 PMC10303517

[20] Charbonnel B. PPAR-alpha and PPAR-gamma activators for type 2 diabetes. Lancet. 2009;374:96–98. doi: 10.1016/S0140-6736(09)61040-0. 19515412

[21] Chen L, Li L, Chen J, Zheng Z, Ren J, Qiu Y. Oleoylethanolamide, an endogenous PPAR-α ligand, attenuates liver fibrosis targeting hepatic stellate cells. Oncotarget. 2015;6(40):42530–42540. doi: 10.18632/oncotarget.6466. 26729705 PMC4767450

[22] Lefebvre P, Chinetti G, Fruchart JC, Staels B. Sorting out the roles of PPAR alpha in energy metabolism and vascular homeostasis. J Clin Invest. 2006;116(3):571–580. 16511589 10.1172/JCI27989 PMC1386122

[23] Dong XC. Sirtuin 6-A key regulator of hepatic lipid metabolism and liver health. Cells. 2023;12(4):663. doi: 10.3390/cells12040663. 36831330 PMC9954390

[24] Wu L, Liu C, Chang DY, Zhan R, Zhao M, Man Lam S, Shui G, Zheng L, Chen M. The attenuation of diabetic nephropathy by annexin A1 via regulation of lipid metabolism through the AMPK/PPARα/CPT1b pathway. Diabetes. 2021;70(10):2192–2203. doi: 10.2337/db21-0050. 34103347

[25] Taschler U, Schreiber R, Chitraju C, Grabner GF, Romauch M, Wolinski H, Haemmerle G, Breinbauer R, Zechner R, Lass A, et al. Adipose triglyceride lipase is involved in the mobilization of triglyceride and retinoid stores of hepatic stellate cells. Biochim Biophys Acta. 2015;1851(7):937–945. doi: 10.1016/j.bbalip.2015.02.017. 25732851 PMC4408194

[26] Kalsi RS, Kreger AM, Saleh M, Yoshida S, Sharma K, Fusco J, Saloman JL, Zhang T, Thomas M, Sehrawat A, et al. Chemical pancreatectomy in non-human primates ablates the acini and ducts and enhances beta-cell function. Sci Rep. 2023;13(1):9113. doi: 10.1038/s41598-023-35820-2. 37277426 PMC10241801

[27] Alhasan H, Terkawi MA, Matsumae G, Ebata T, Tian Y, Shimizu T, Nishida Y, Yokota S, Garcia-Martin F, Abd Elwakil M, et al. Inhibitory role of annexin A1 in pathological bone resorption and therapeutic implications in periprosthetic osteolysis. Nat Commun. 2022;13(1):3919. doi: 10.1038/s41467-022-31646-0. 35798730 PMC9262976

[28] Yang W, Ling X, He S, Cui H, Yang Z, An H, Wang L, Zou P, Chen Q, Liu J, et al. PPARα/ACOX1 as a novel target for hepatic lipid metabolism disorders induced by per- and polyfluoroalkyl substances: an integrated approach. Environ Int. 2023;178:108138. doi: 10.1016/j.envint.2023.108138. 37572494

[29] Schulze RJ, Ding WX. Lipid droplet dynamics in alcoholic fatty liver disease. Liver Res. 2019;3(3–4):185–190. doi: 10.1016/j.livres.2019.09.002. 33664985 PMC7928432

[30] Morley M, Molony CM, Weber TM, Devlin JL, Ewens KG, Spielman RS, Cheung VG. Genetic analysis of genome-wide variation in human gene expression. Nature. 2004;430(7001):743–747. doi: 10.1038/nature02797. 15269782 PMC2966974

[31] Chick JM, Munger SC, Simecek P, Huttlin EL, Choi K, Gatti DM, Raghupathy N, Svenson KL, Churchill GA, Gygi SP. Defining the consequences of genetic variation on a proteome-wide scale. Nature. 2016;534(7608):500–505. doi: 10.1038/nature18270. 27309819 PMC5292866

