# Supplementary material for: Annexin A1 exacerbates islet stellate cell activation by regulating triglyceride catabolism via the PPARα/ACOX1/CYP4a pathway
Source: Islets. 2026 Feb 22;18(1):2633793. doi: 10.1080/19382014.2026.2633793 (PMC12928626; doi:10.1080/19382014.2026.2633793)
Supplement: supplementary materials Table 3 Instruments.docx — supplementary materials Table 3: Instruments.docx [file KISL_A_2633793_SM7449.docx]

| Microplate Reader | TECAN Switzerland |
| --- | --- |
| Gradient PCR Amplifier | Thermo Fisher Scientific USA |
| Real-Time Fluorescent Quantitative PCR Instrument | Thermo Fisher Scientific USA |
| Benchtop High-Speed Refrigerated Centrifuge | Eppendorf Germany |
| Micro Vortex Mixer | Thermo Fisher Scientific, USA |
| Magnetic Stirrer | Thermo Fisher Scientific, USA |
| Vertical Electrophoresis Unit | Thermo Fisher Scientific |
| Blot Transfer System | Thermo Fisher Scientific |
| Fluorescence Microscope | Olympus, Japan |
| Chemiluminescence Imager | Bio-Rad Technologies, Inc. |
| Ultramicrotome | AIRTECH, Japan |
| Paraffin Embedding Machine | Wuhan Junjie Electronics Co., Ltd. |
| Electronic Balance | Mettler Toledo, Switzerland |
| Upright Optical Microscope | AIRTECH, Japan |
