# Supplementary figures and images for: Annexin A1 exacerbates islet stellate cell activation by regulating triglyceride catabolism via the PPARα/ACOX1/CYP4a pathway
Source: Islets. 2026 Feb 22;18(1):2633793. doi: 10.1080/19382014.2026.2633793 (PMC12928626; doi:10.1080/19382014.2026.2633793)

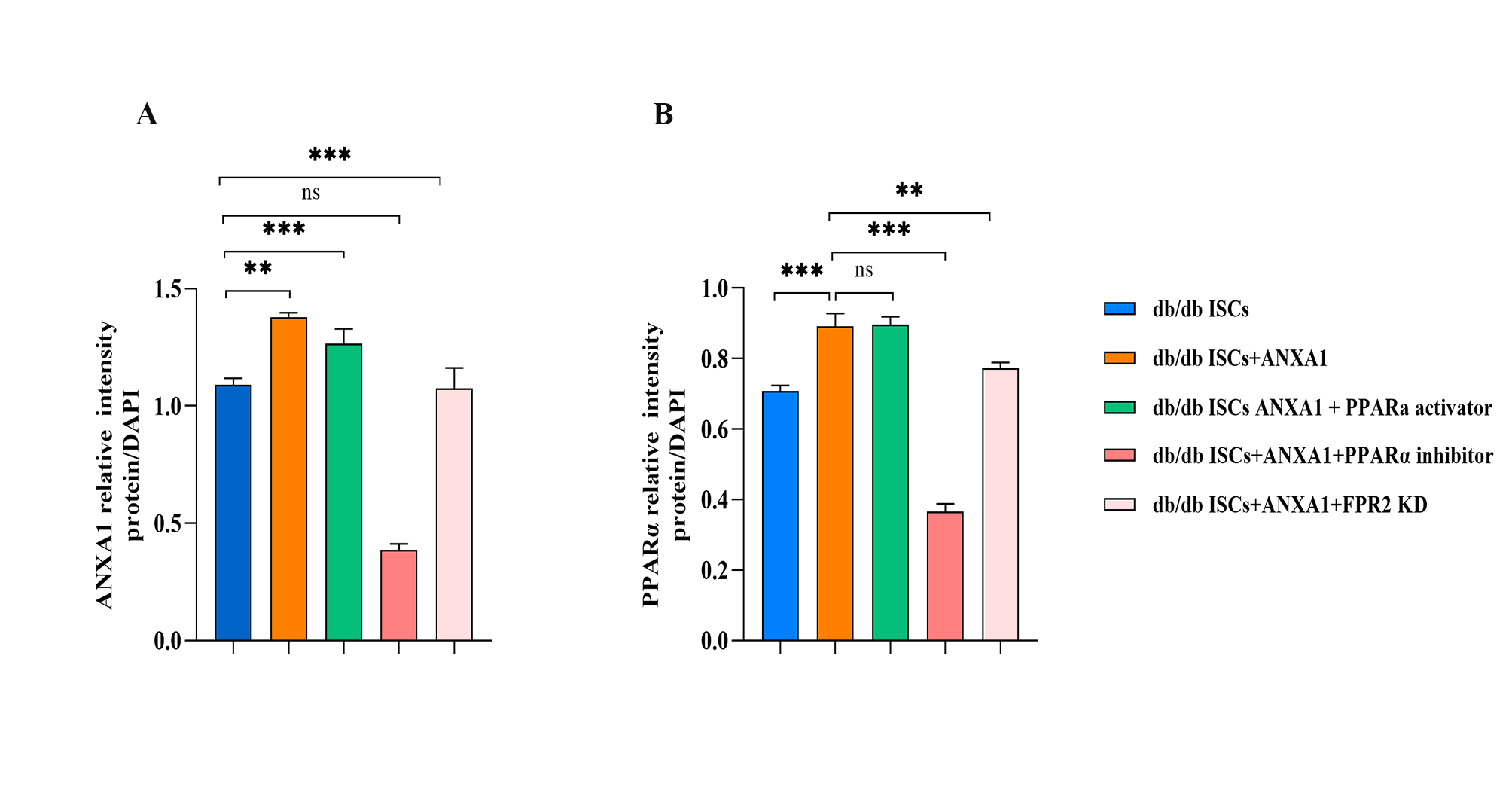

Supplement: supplementary materials 4 Fig 6 quantitative analysis.tif — supplementary materials 4: Fig 6 quantitative analysis.tif [file KISL_A_2633793_SM7447.tif]
