# Supplementary material for: Annexin A1 exacerbates islet stellate cell activation by regulating triglyceride catabolism via the PPARα/ACOX1/CYP4a pathway
Source: Islets. 2026 Feb 22;18(1):2633793. doi: 10.1080/19382014.2026.2633793 (PMC12928626; doi:10.1080/19382014.2026.2633793)

From left to right:

1. Marker
2. db/db ISCs NC
3. db/db ISCs + ANXA1
4. db/db ISCs + ANXA1 +PPARa activator
5. db/db ISCs + ANXA1+PPARa inhibitor
6. db/db ISCs FPR2 KD+ANXA1

GAPDH

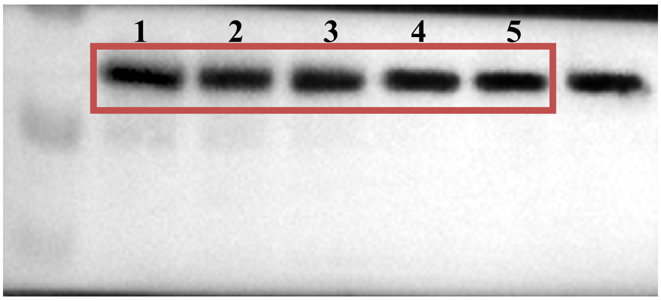

36KDa

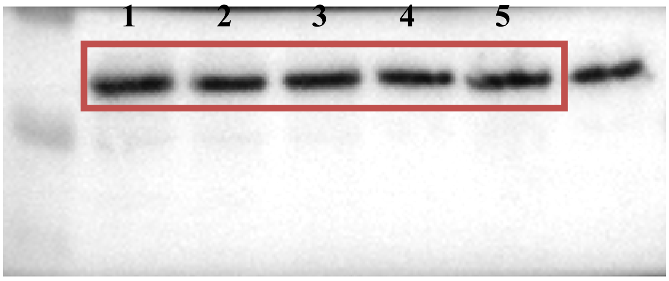

36KDa

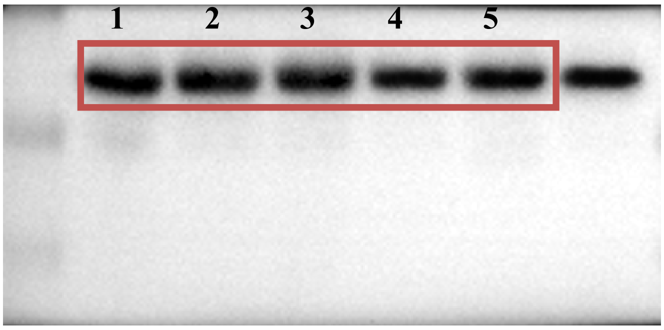

36KDa

FPR2

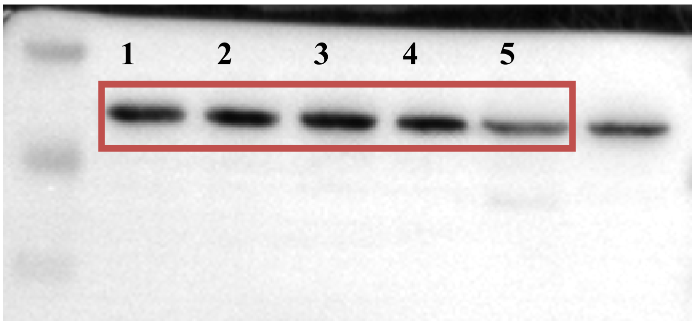

38KDa

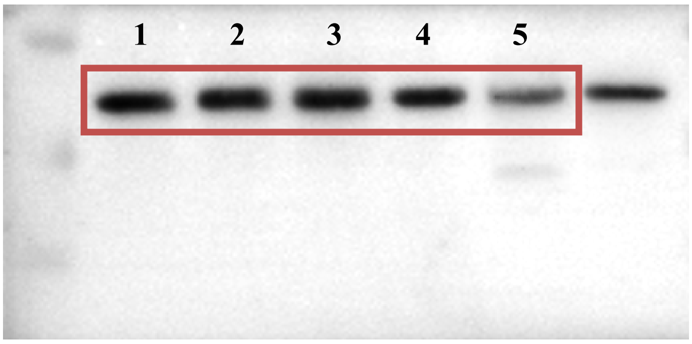

38KDa

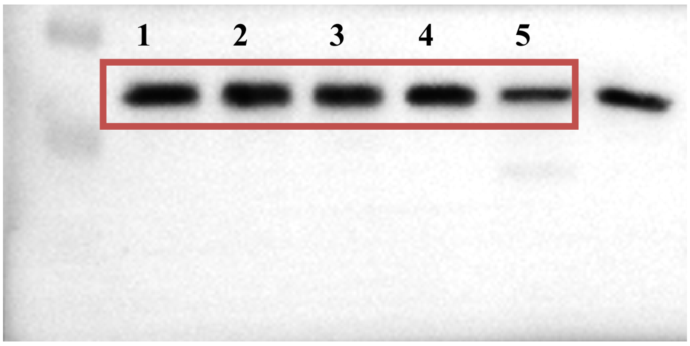

38KDa

ATGL

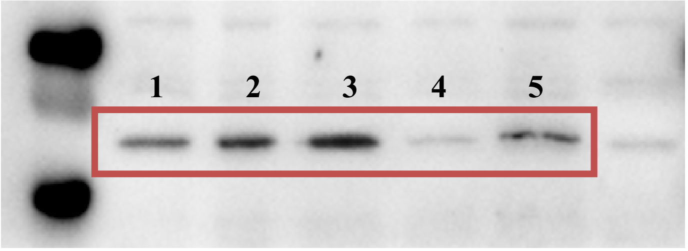

60KDa

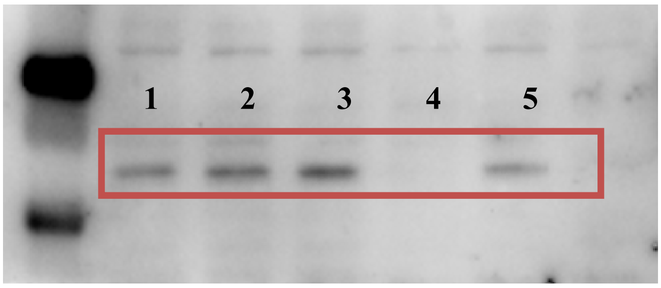

60KDa

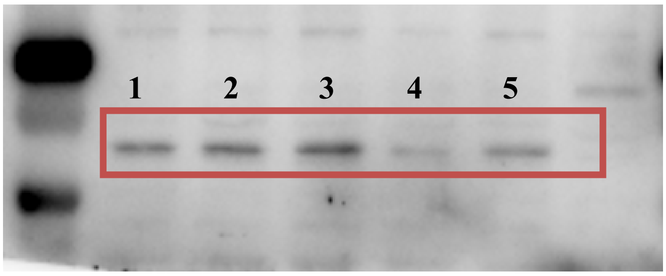

60KDa

HSL

1 2 3 4 5

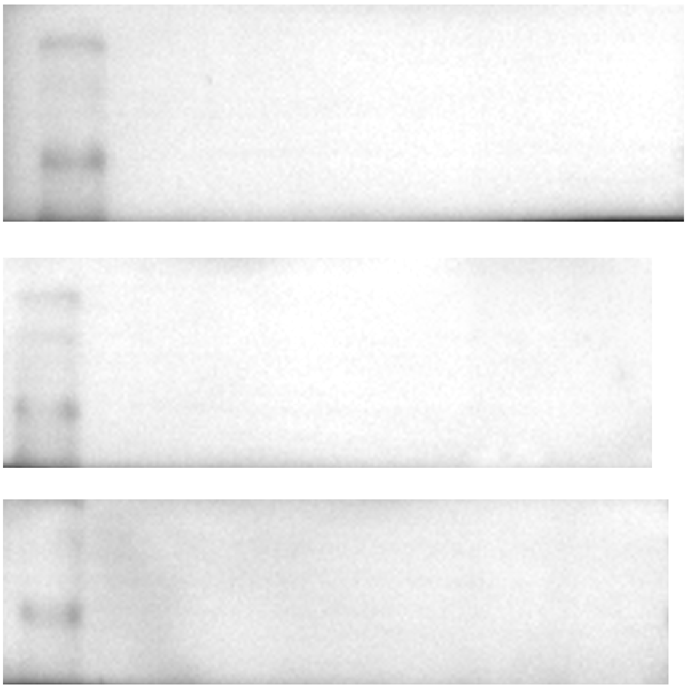

DGAT2

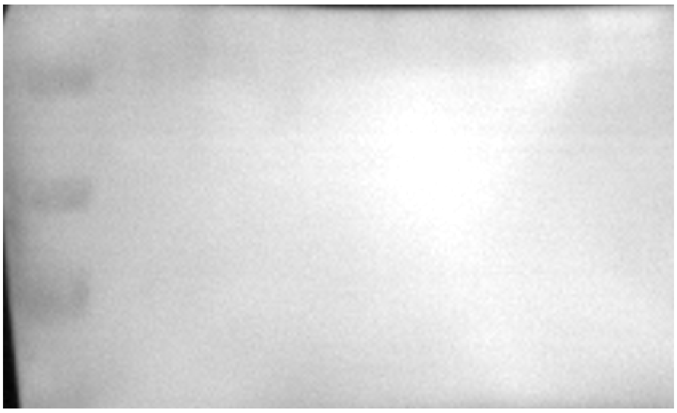

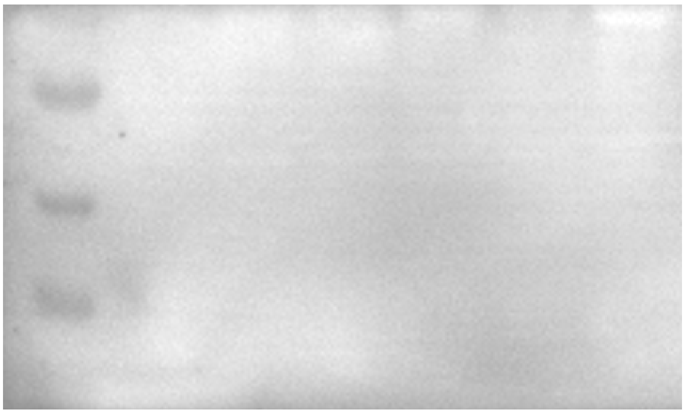

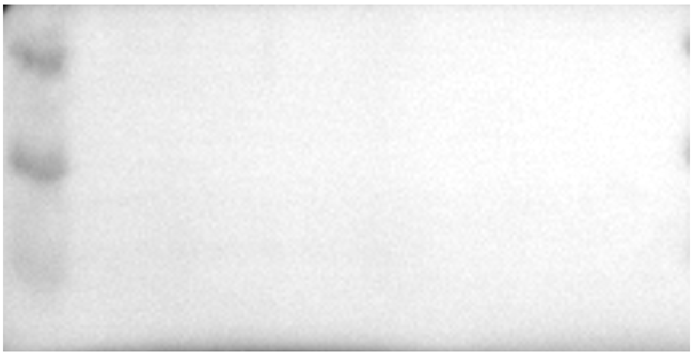

PPARa

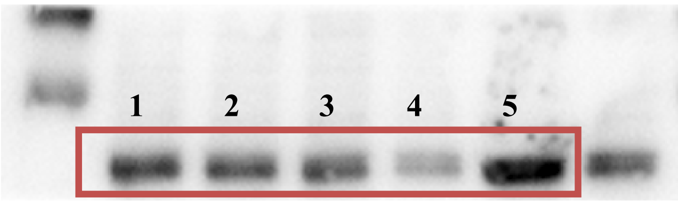

56KDa

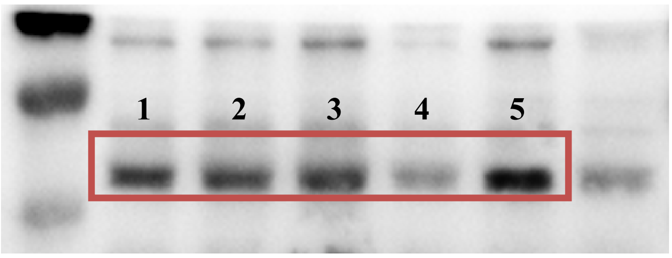

56KDa

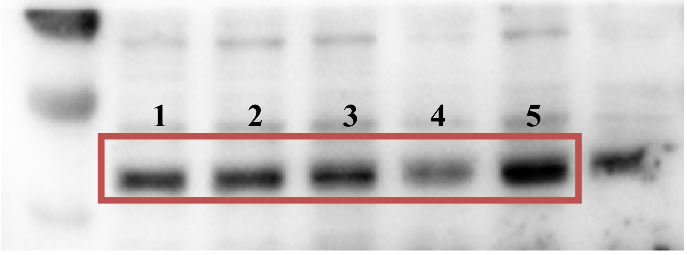

56KDa

CPT1B

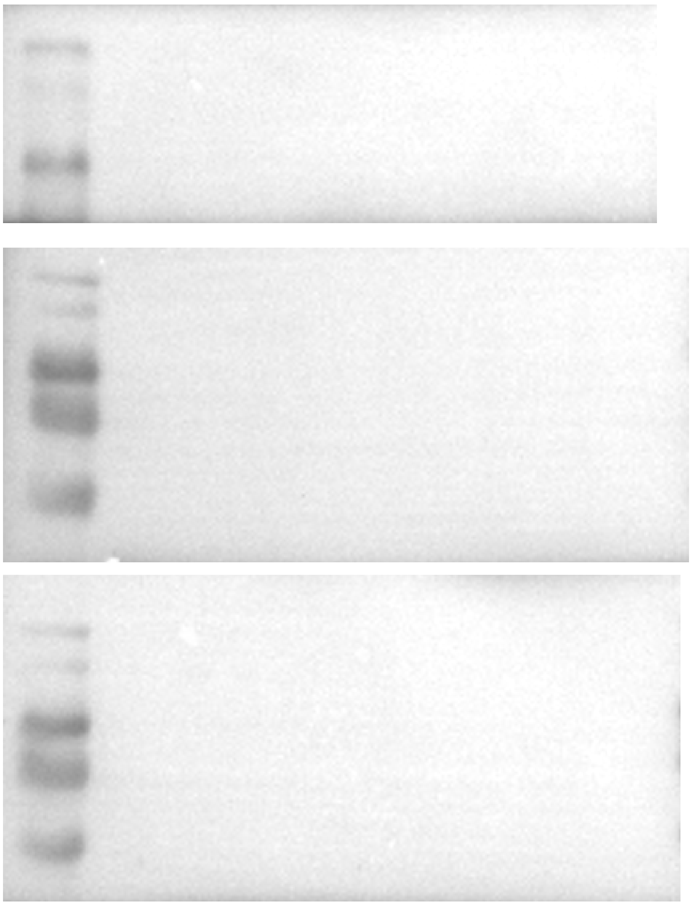

DGAT1

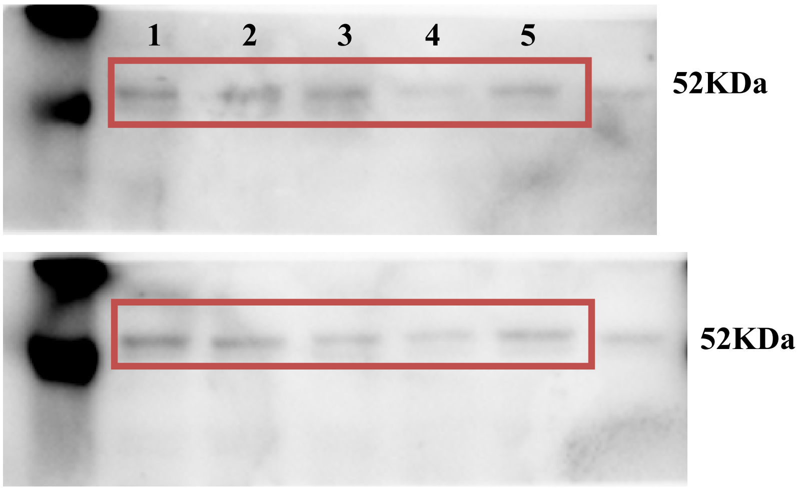

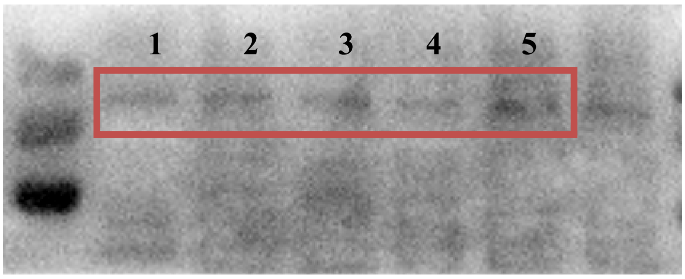

52KDa

ACOX1

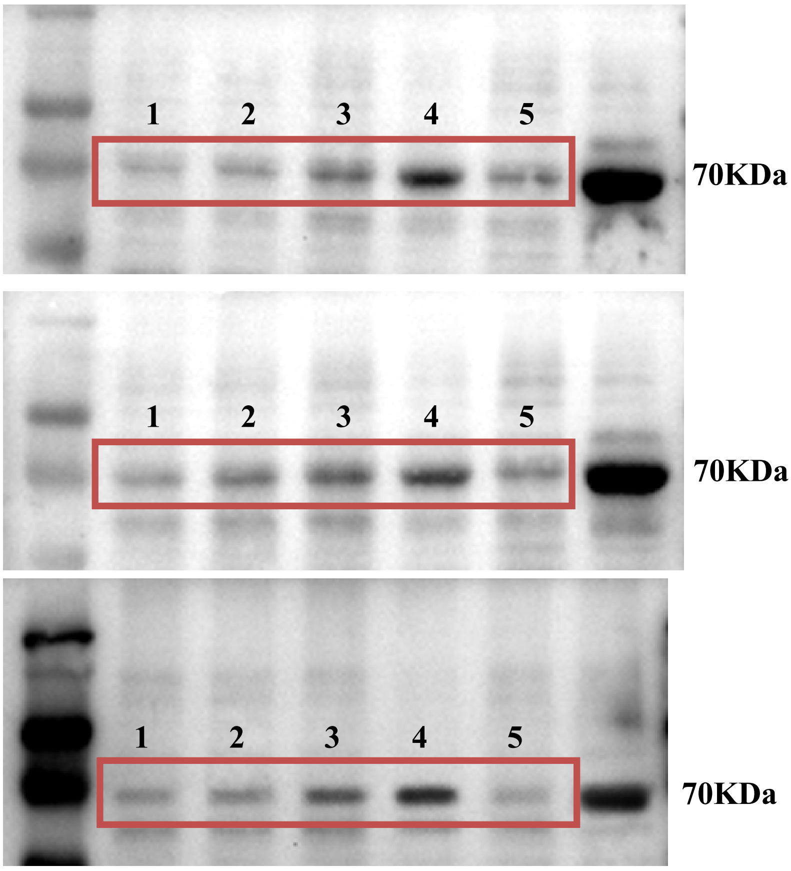

70KDa

70KDa

70KDa

CYP4A

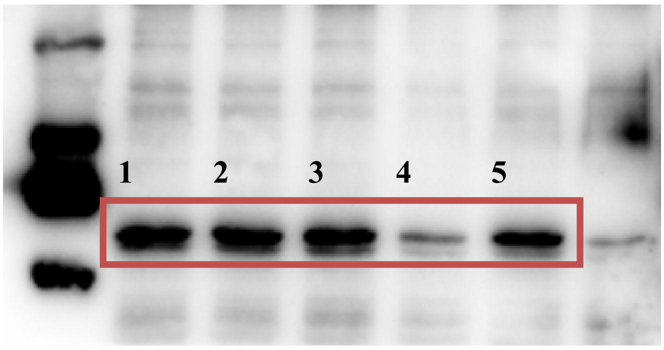

50KDa

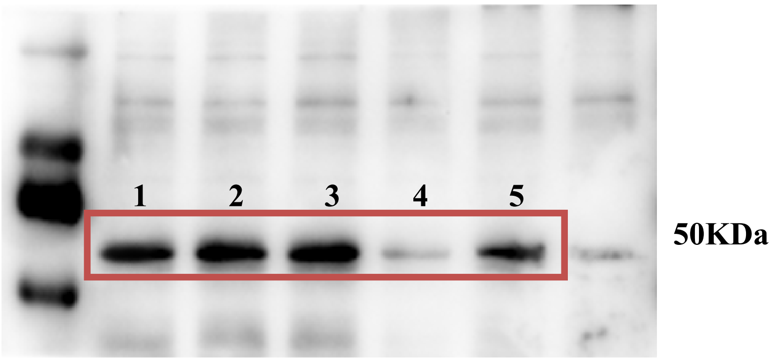

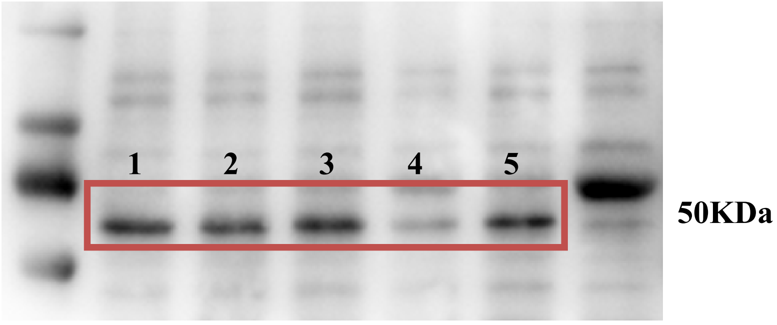

Co-IP

Mouse IgG (PPARa)

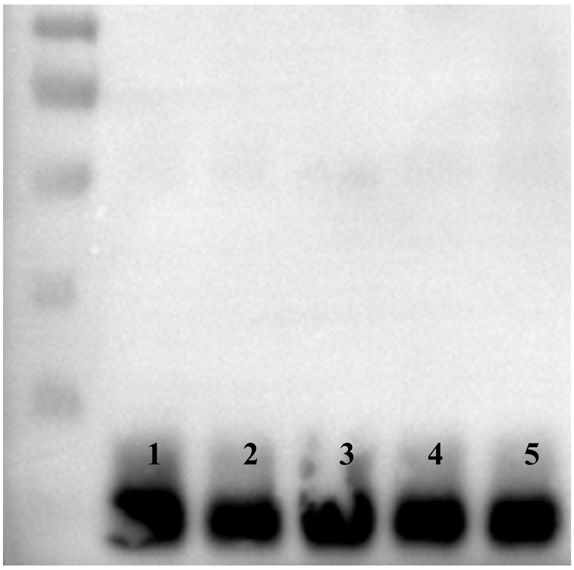

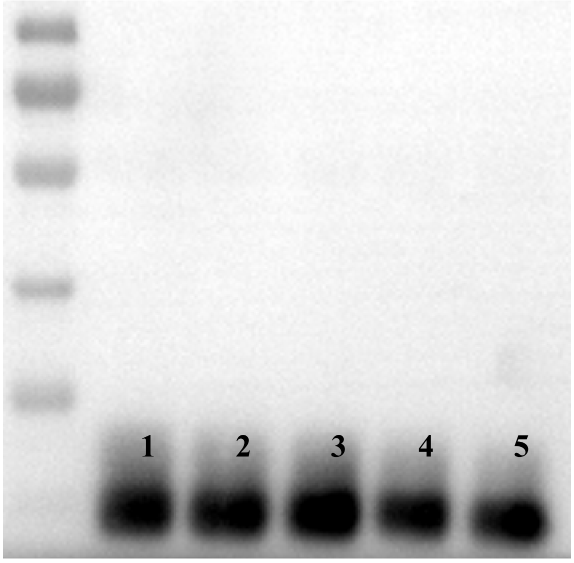

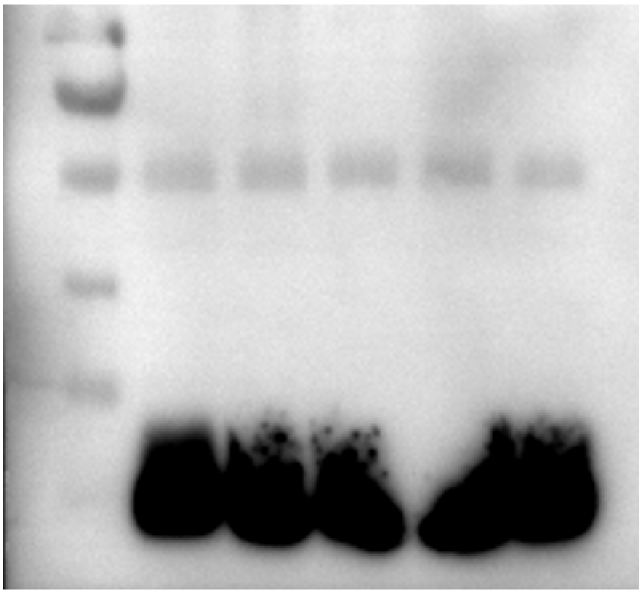

Mouse IgG (ANXA1)

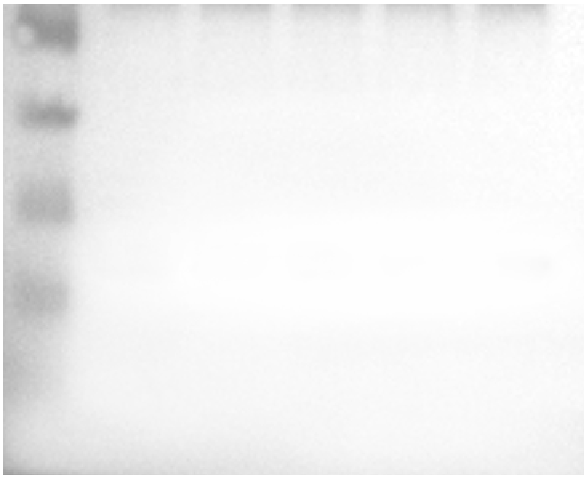

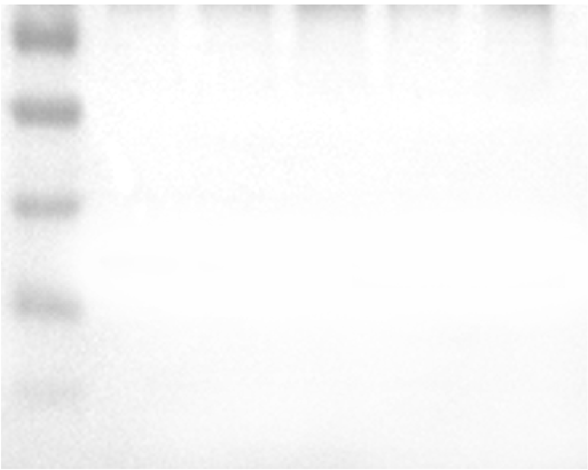

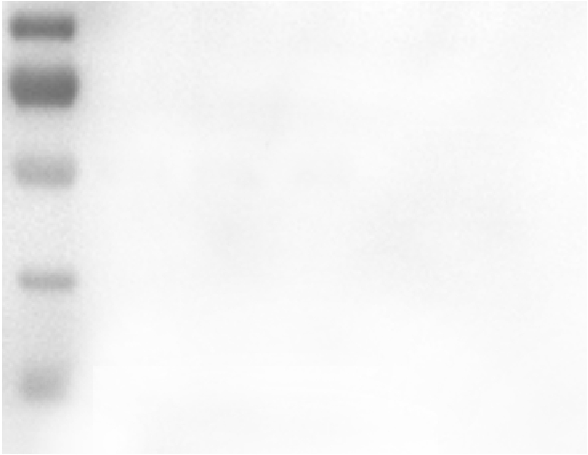

Rabbit IgG (ANXA1)

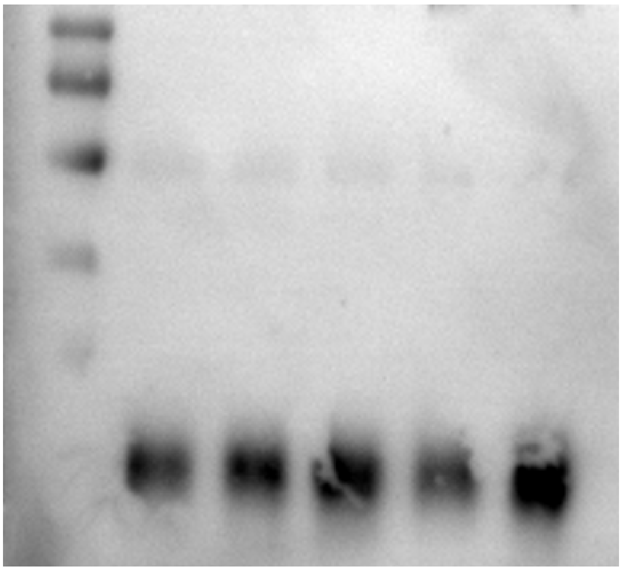

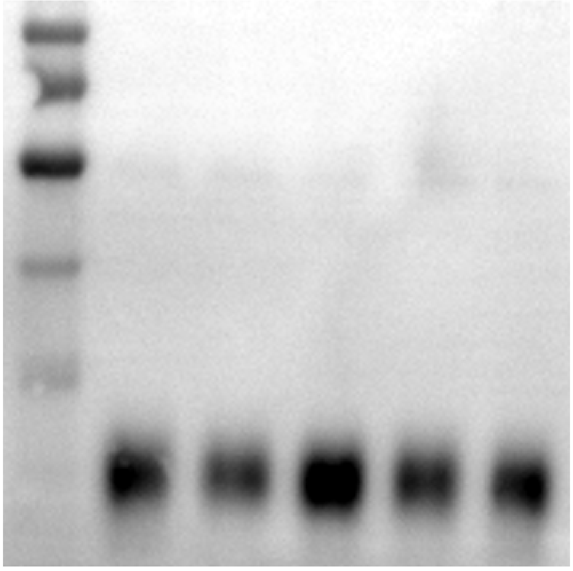

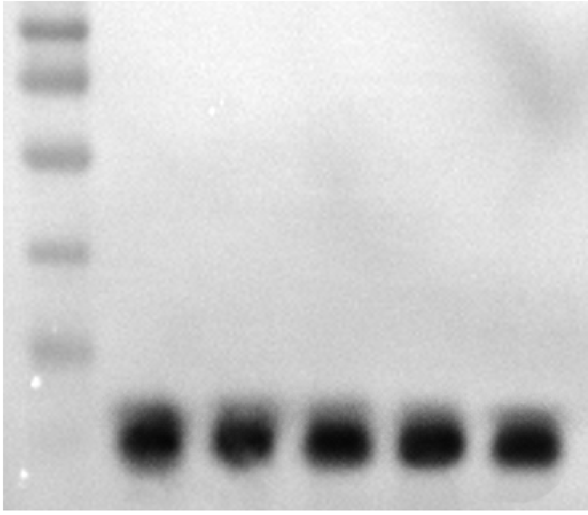

Rabbit IgG (PPARa)

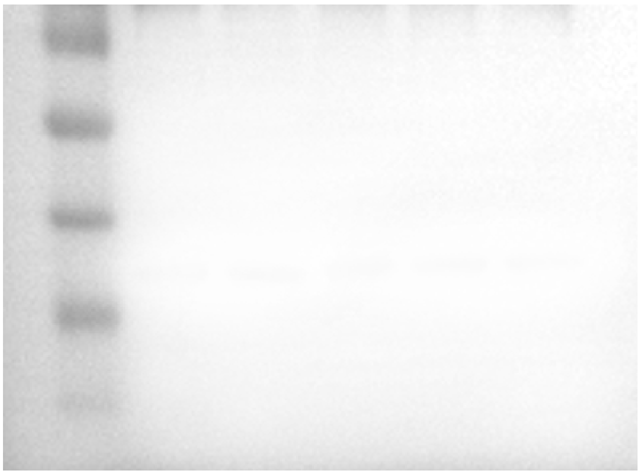

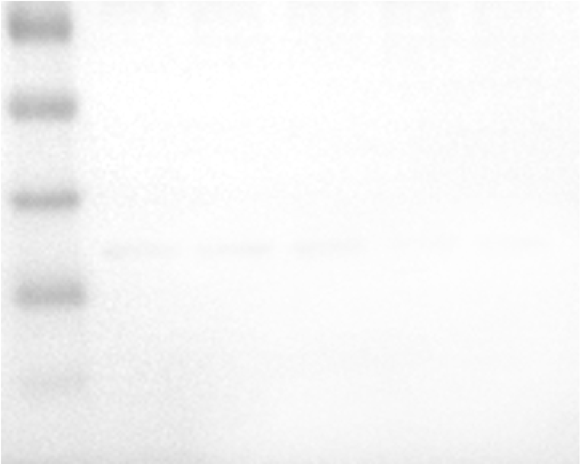

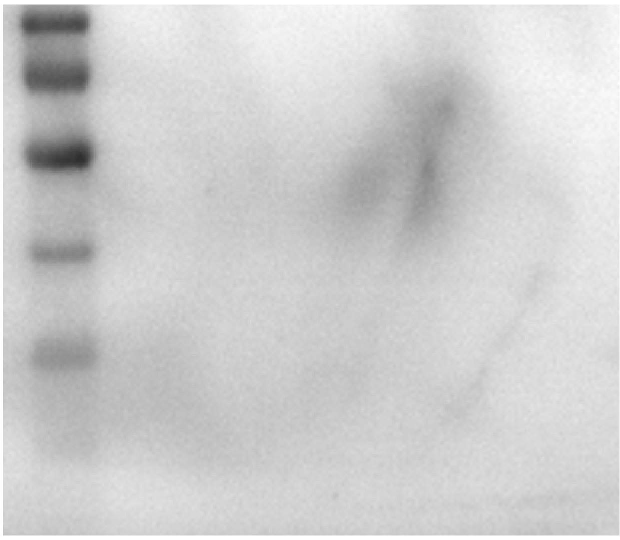

ANXA1 CoIP
PPARa

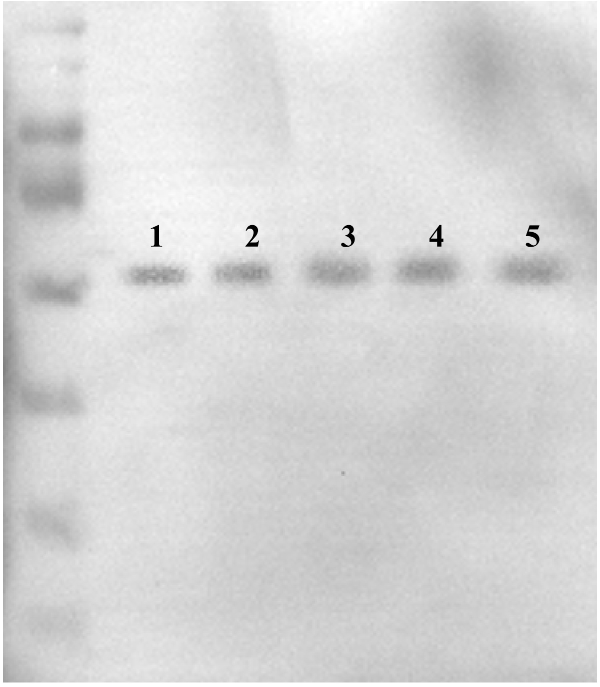

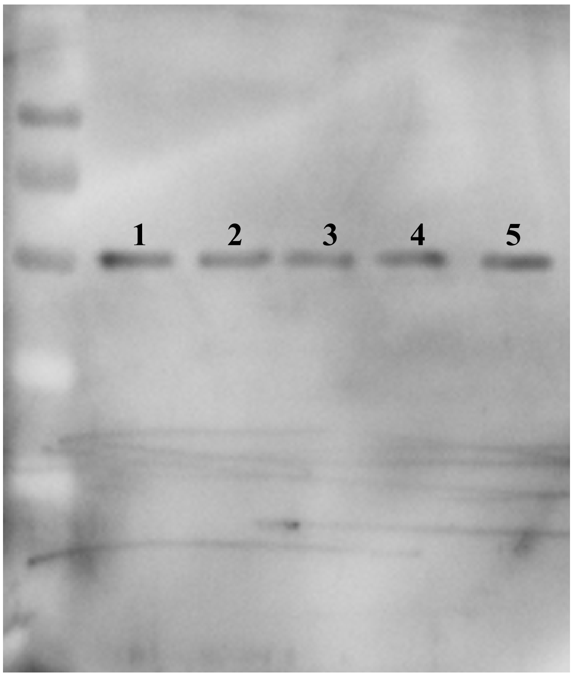

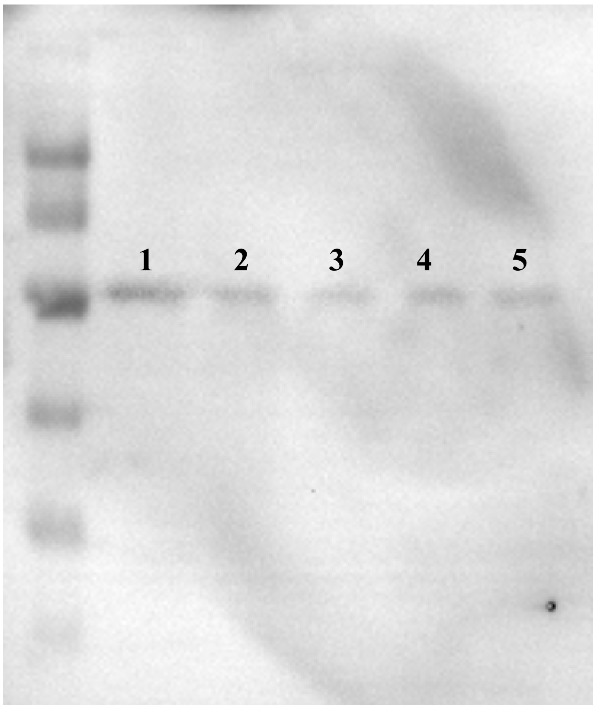

ANXA1

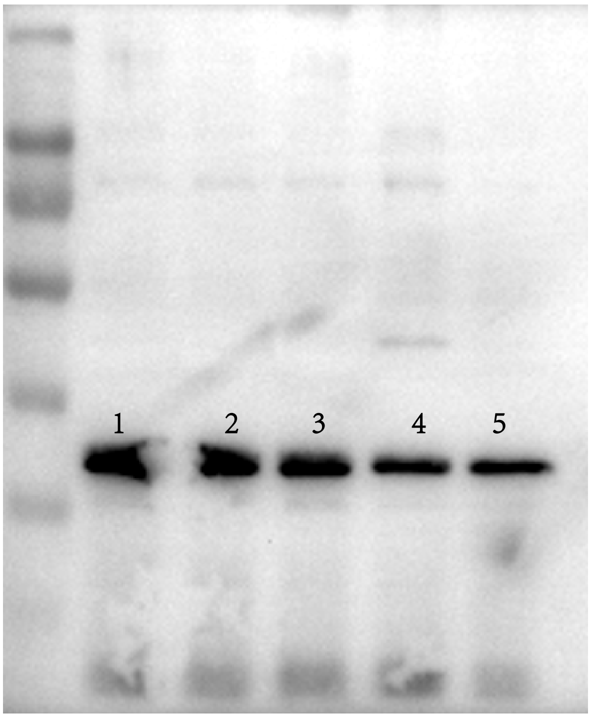
1
2
3
4
5
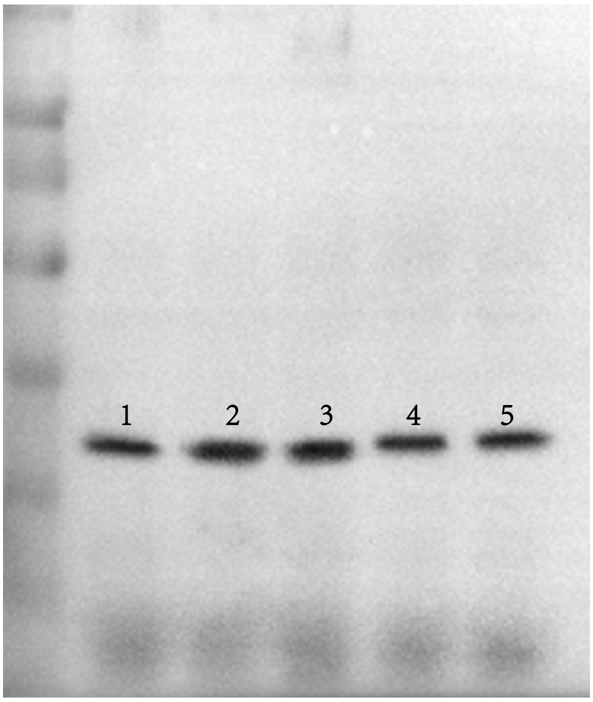
1
2
3
4
5

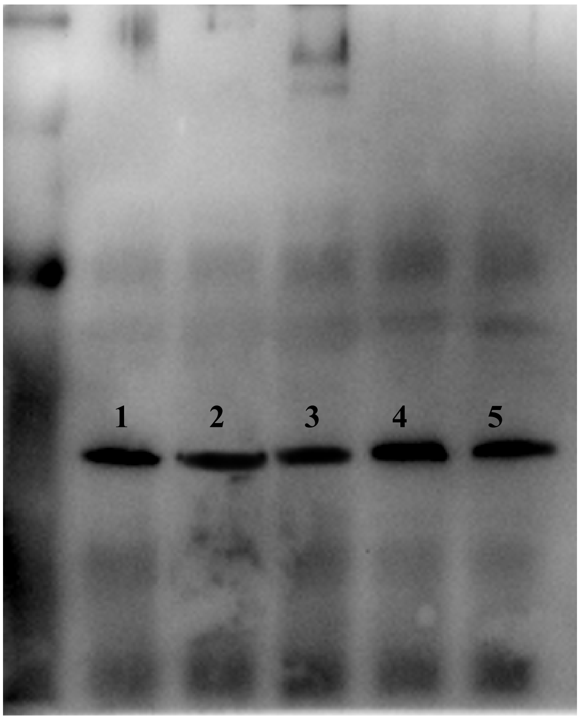

PPARa CoIP
PPARa

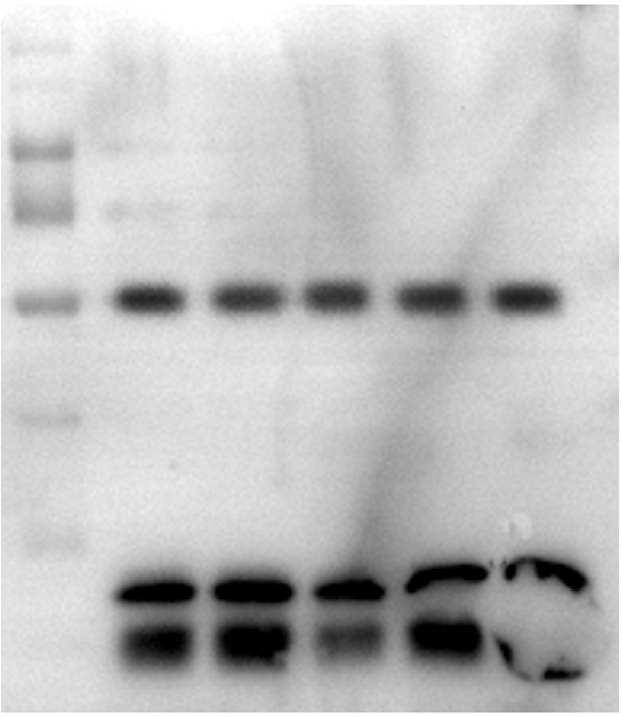

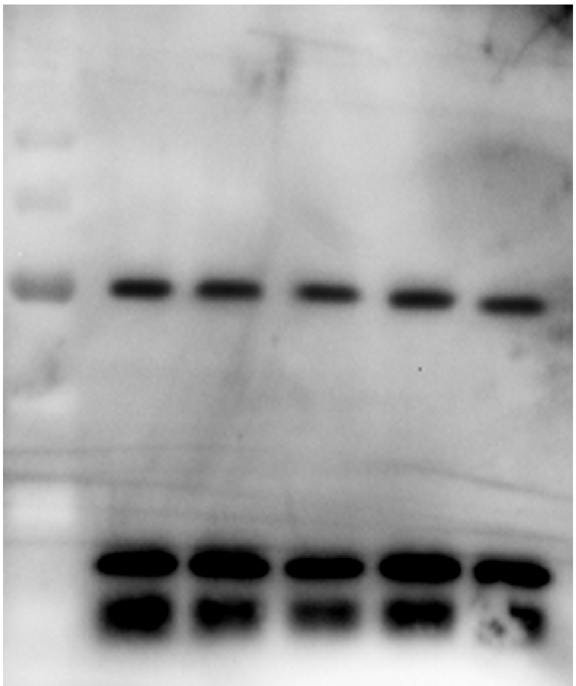

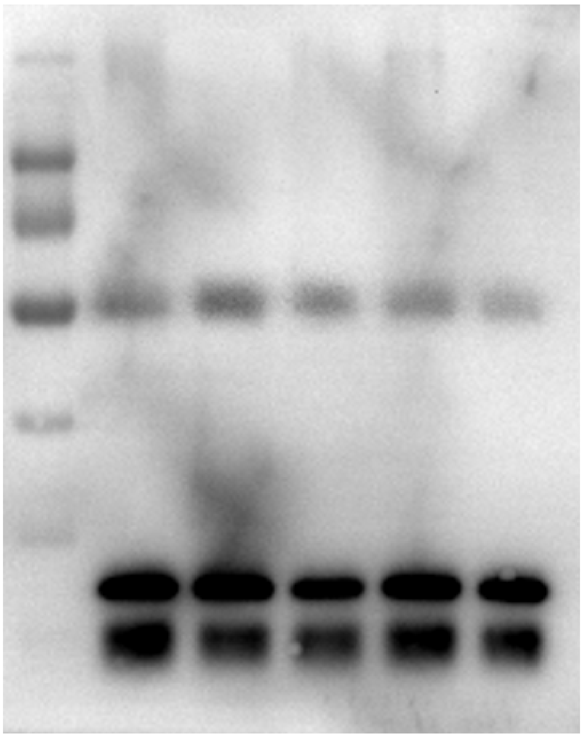

ANXA1

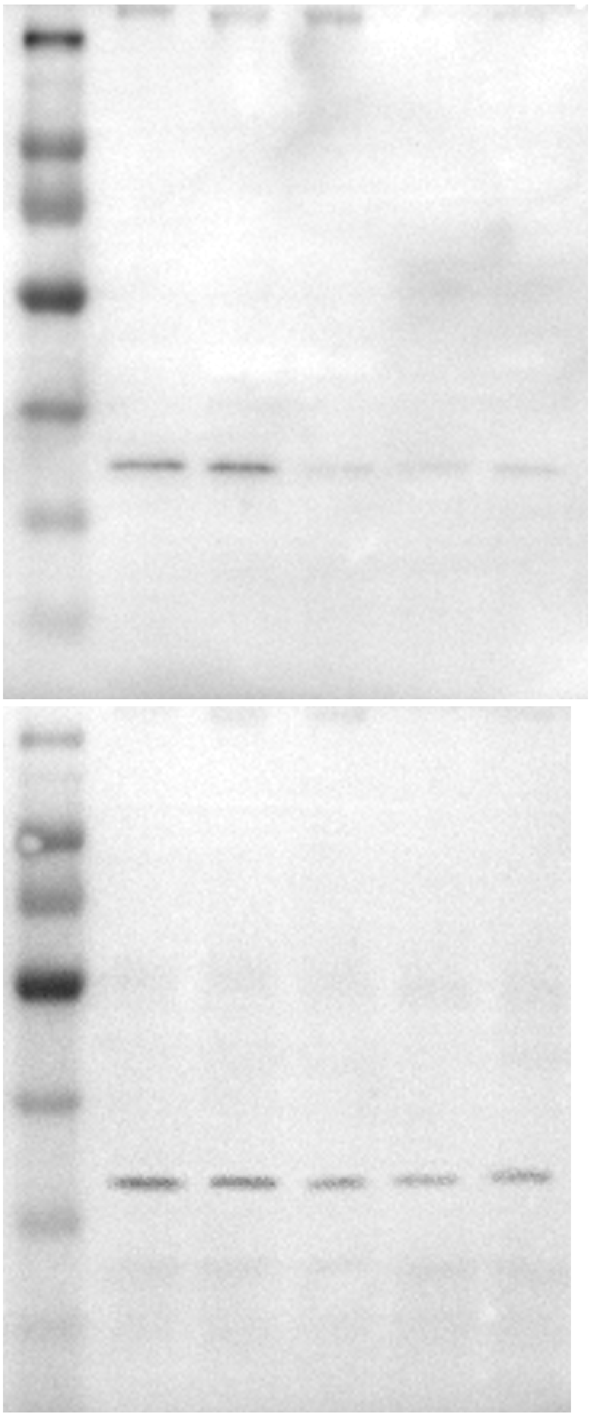

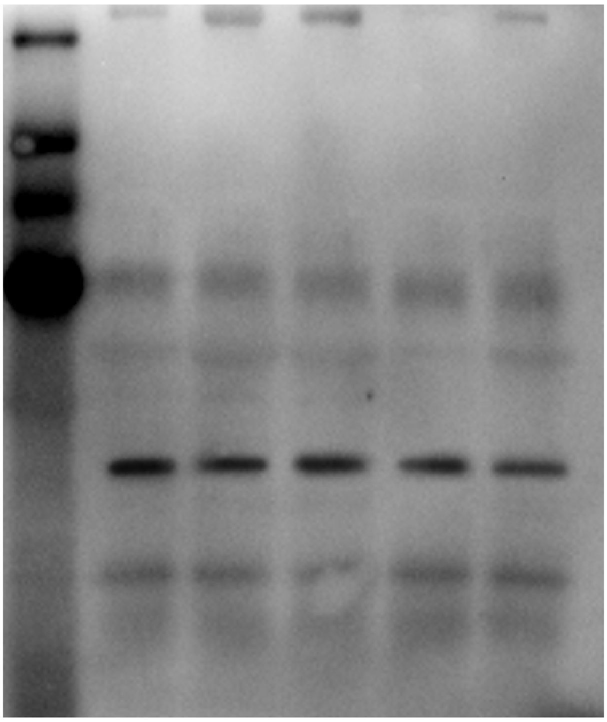

WB
GAPDH

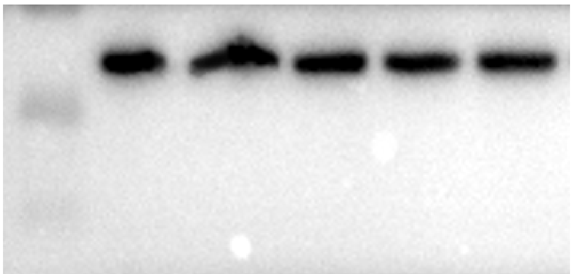

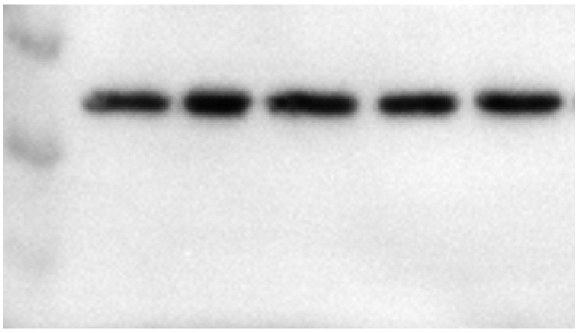

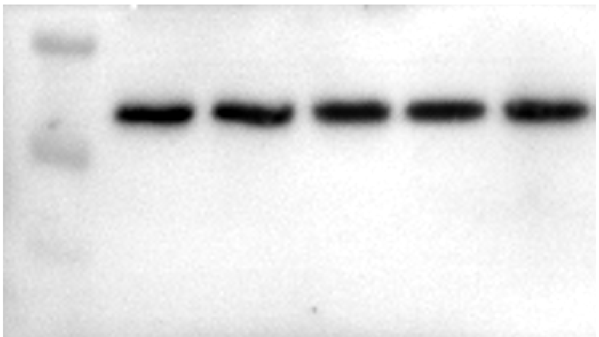

ANXA1

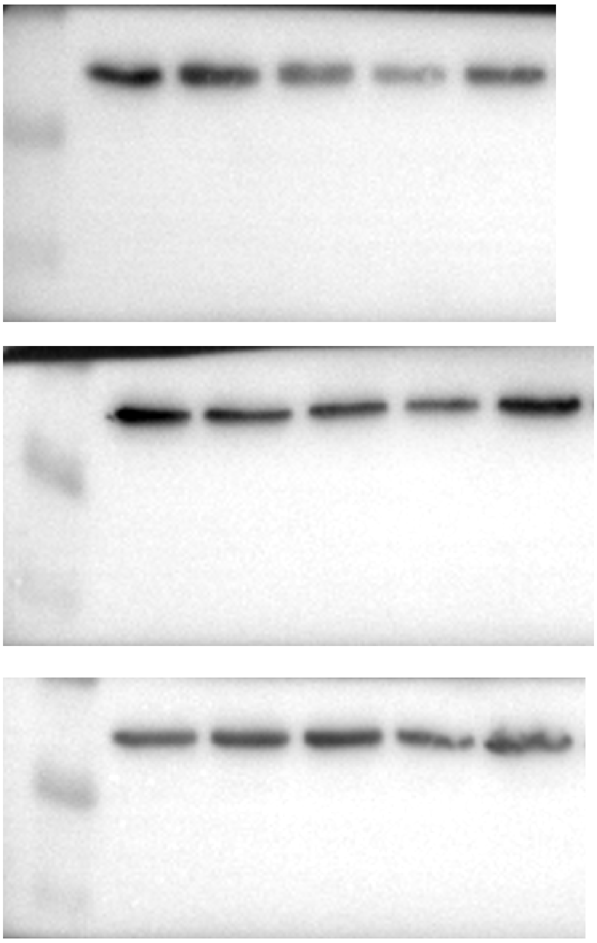

PPARa

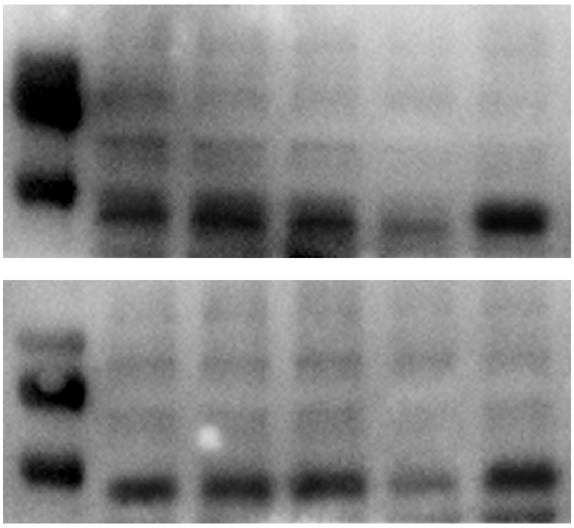

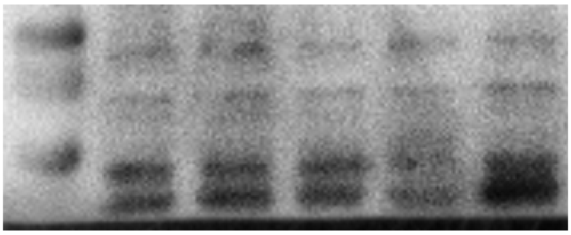

Supplement: supplementary materials_raw_images updated.pdf [file KISL_A_2633793_SM7448.pdf]
